# Analysis of small-angle scattering data of interacting ellipsoids using pair distance distribution functions

**DOI:** 10.1107/S1600576726004383

**Published:** 2026-06-15

**Authors:** Gerhard Fritz-Popovski

**Affiliations:** a Technical University Leoben Department of Physics, Mechanics and Electrical Engineering Chair of Physics 8700LeobenAustria; Argonne National Laboratory, USA

**Keywords:** small-angle scattering, pair distance distribution functions, ellipsoidal particles, generalized indirect Fourier transformation

## Abstract

Pair distance distribution functions (PDDFs) similar to those obtained by indirect Fourier transformation of small-angle scattering data from interacting prolate and oblate ellipsoids have been simulated using a Monte Carlo approach. Interactions were modelled with a square-well or square-wall potential combined with a non-overlapping core, and spanned from a maximum attraction of −1.5 *kT* per pair of particles to a repulsion of +1.5 *kT*. Volume fractions ranged from 1% to 10%, with an extension to 20% for systems in which particles interacted solely via non-overlapping cores, without further attraction or repulsion. The data were also transformed into scattering curves and analysed using the generalized indirect Fourier transformation (GIFT). The results show that rules of thumb for interpreting PDDFs can be misleading. GIFT removes most interaction effects and allows correct identification of the ellipsoidal shape; however, axis lengths are often underestimated. Attractive interactions are more challenging than repulsive ones and may go unnoticed without additional information.

## Abstract 2

Pair distance distribution functions of interacting ellipsoidal particles can be hard to interpret. This difficulty can be circumvented partially by the generalized indirect Fourier transformation.

## 1. Introduction

Small-angle scattering has firmly established itself as a helpful method for investigating structures within the size range of several nanometres (Guinier & Fournet, 1955 ▸; Glatter & Kratky, 1982 ▸; Feigin & Svergun, 1987 ▸; Lindner & Zemb, 1991 ▸; Brumberger, 1995 ▸; Lindner & Zemb, 2002 ▸; Glatter, 2018 ▸). This encompasses, for instance, inorganic nanoparticles (Li *et al.*, 2016 ▸), self-assembled structures (Chen, 1986 ▸), proteins (Trewhella, 2022 ▸), polymers (Chu & Hsiao, 2001 ▸; Wei & Hore, 2021 ▸) and non-particulate structures (Finnefrock *et al.*, 2003 ▸).

The experimental output consists of scattering patterns or curves, which require further interpretation to extract the desired structural information. This can involve calculating global parameters from the scattering curves (Porod, 1982 ▸), although more commonly the observed data are explained by developing an appropriate model. A typical approach involves comparing theoretically calculated scattering patterns directly with experimental data (Pedersen, 2002*a* ▸). Alternatively, the scattering pattern can be transformed into its real-space counterpart, serving as an intermediate step. Several such real-space analogues are employed, including the autocorrelation function (Debye & Bueche, 1949 ▸) and the chord length distribution (Guinier & Fournet, 1955 ▸); however, the pair distance distribution function (PDDF) (Porod, 1949 ▸) is arguably the most widely used. These functions retain the same information as the original scattering pattern but benefit from being expressed in real space, enabling direct interpretation in terms of distances. This facilitates the construction of a suitable model, as many features become immediately interpretable in terms of particle size, shape and internal structure (Glatter, 1979 ▸; Fritz & Bergmann, 2004 ▸). The computation of such real-space functions is likewise straightforward, relying on the method of indirect Fourier transformation (IFT) (Glatter, 1977*a* ▸; Glatter, 1977*b* ▸; Moore, 1980 ▸; Svergun *et al.*, 1988 ▸; Hansen & Pedersen, 1991 ▸).

When the system under investigation consists of particles, a typical objective is to derive information about an individual particle. However, interaction effects may introduce complexity. In the simplest case, the scattering curve can be described as the product of the particle form factor and a structure factor (Zernike & Prins, 1927 ▸; Kotlarchyk & Chen, 1983 ▸). The form factor encapsulates the sought-after information about the individual particle, while the structure factor reflects the particle concentration and spatial arrangement. Consequently, transforming the scattering curve into real space yields a function that represents a convolution of the PDDF of the individual particle with the real-space analogue of the particle arrangement (Fritz-Popovski *et al.*, 2011 ▸).

The separation of intra- and interparticle contributions can be achieved through the generalized indirect Fourier transformation (GIFT) (Brunner-Popela & Glatter, 1997 ▸; Bergmann *et al.*, 2000 ▸; Fritz & Glatter, 2006 ▸). Unlike other transformation methods, this approach is not entirely model free, as it necessitates the use of a model for the structure factor. Nevertheless, it remains a robust technique, as the particle arrangement—and, by extension, the structure factor—is relatively insensitive to the finer details of the interaction potential (Hansen & McDonald, 2005 ▸).

The initial step of a GIFT evaluation, however, is always a transformation into real space without making any assumptions regarding particle interactions. The resulting real-space curve can often be directly interpreted in terms of probable particle size and shape, thereby excluding a wide range of potential models. Furthermore, it provides valuable insights into the type of interaction model that would be most appropriate for a subsequent GIFT calculation, enhancing the precision of the analysis.

The PDDFs of globular particles are probably the most well understood, owing to the availability of suitable structure factor models (Percus & Yevick, 1958 ▸; Madden & Rice, 1980 ▸; Hansen & Hayter, 1982 ▸; Rogers & Young, 1984 ▸; Zerah & Hansen, 1986 ▸; Klein & D’Aguanno, 2015 ▸; Brunner-Popela & Glatter, 1997 ▸). These models enable swift simulations and render the corresponding real-space data readily accessible. However, this may pose a challenge, as PDDFs of interacting particles that deviate from spherical symmetry could be misinterpreted. Researchers may be inclined to describe them in a manner analogous to globular particles, leading to potential inaccuracies.

This study seeks to enhance our understanding of the subject by examining the PDDFs of ellipsoids of revolution across a range of concentrations and interaction potentials. The spatial functions are derived through Monte Carlo simulations, and the findings are compared with experimentally determined PDDFs of protein solutions, using lysozyme as a model system for non-spherical particles.

The discussion of the pair distance distribution functions proceeds in two stages, corresponding to the first two interpretative steps in the GIFT procedure. First, we consider the PDDF of the interacting system, equivalent to that obtained by IFT. On the basis of this PDDF, we then select a model for the effective structure factor used in the GIFT analysis to remove interaction effects; such models typically assume spherical particles, because interactions often obscure details of particle shape. We then assess the resulting PDDFs, highlighting the limitations introduced by the chosen structure factor models.

One of these limitations is clearly the lack of dedicated effective structure factor models provided by GIFT. Typically, the decoupling approximation (Kotlarchyk & Chen, 1983 ▸) or the local monodisperse approximation (Pedersen, 1994 ▸) is used to compute effective structure factors of such particles based on a size distribution of spheres, but they are not available in the current version of GIFT. The most flexible model incorporated is the averaged structure factor model (Brunner-Popela & Glatter, 1997 ▸). Since it has been used successfully for non-spherical particles (Glatter *et al.*, 2000 ▸), this model will be used for the GIFT evaluations.

Both individual particles and systems of interacting particles can be described by PDDFs. In this work, we employ both formulations. We write PDDF_i_ and *i*(*r*) when interparticle effects are present. When such effects are removed and only single-particle effects are expected, we write PDDF_p_ and *p*(*r*).

## 2. Experimental

### 2.1. Simulations

The PDDF_i_s for homogeneous spheres (radius 2 nm), oblate ellipsoids of revolution (semi-axes of 2, 2 and 1 nm) and prolate ellipsoids (semi-axes of 2, 1 and 1 nm) were simulated using a Monte Carlo method (Pedersen, 2002*b* ▸). The particles were arranged to prevent overlap, and an interaction layer of 0.5 nm thickness surrounding the ellipsoids was included to account for an additional interaction potential. The strength of this potential was proportional to the overlapping volumes of the interaction layers between two particles and scaled such that the maximum overlap corresponded to an energy contribution β. The parameter β was varied as follows: β = −1.5 *kT*, β = −1.0 *kT*, β = −0.5 *kT*, β = 0.0, β = +0.5 *kT*, β = +1.0 *kT* and β = +1.5 *kT*. For spheres, β was always set to 0.0, as they primarily served as a reference to compare results with simpler structure factor models.

The particles were distributed within a simulation box employing periodic boundary conditions, with the side length *a* of the box adjusted to achieve the specified volume fraction. The total number of particles simulated was given by *N*φ, where φ represents the volume fraction and the value *N* = 600 was used. Volume fractions of φ = 0.010, φ = 0.025, φ = 0.050 and φ = 0.100 were simulated for all particle types. Additionally, simulations were performed for φ = 0.150 and φ = 0.200 for particles with β = 0.0.

Particles were placed at random within the simulation box. Each particle was then moved to a new random position with a random orientation. This new position was checked in two ways. First particles were not allowed to overlap. If the centre-to-centre distance to any of the other particles in the system was less than twice the length of the longest axis, 10^5^ random points were created within a cuboid that had its axes parallel to the axes of the coordinate system and was sized to contain both ellipsoids. If any of these points was within both ellipsoids, the new position of the particle was rejected. However, if no overlap occurred, a similar procedure was used to compute the overlapping volume of the interaction layers by Monte Carlo integration. The change in interaction energy due to this new position of the particle was calculated. Then the proposed move was accepted or rejected according to the Boltzmann criterion. If one of the two rejection criteria applied, another random position and orientation were tested. This was repeated for each particle, until a new position was found and accepted. After all particles had been moved, the procedure was repeated using a different random sequence of particles. This equilibration cycle was performed 20 times, with the total energy monitored throughout to check for equilibrium.

The resulting configuration was used to compute one PDDF_i_. Thereafter, each particle was moved again to a new random position in a new random sequence and a further PDDF_i_ was calculated. The entire workflow, starting from the initial placement of the particles, was executed on at least eight cores in parallel. In total, *n*_C_ = 8000 configurations were simulated for each system.

The PDDF_i_ of a single configuration was then determined by sampling *n*_P_ = 10^6^ pairs of points, where one point was chosen randomly within the simulation box and the second within a sphere of radius *fa* centred on the first point, with *f* = 0.6 being employed. The contrast 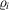 of a point *i* was defined as (1 − φ) if the point lay within a particle and −φ if it lay outside a particle. A contribution of 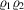 was then added to the appropriate bin of a histogram corresponding to the distance between the two points. The number of bins in the histograms was *n*_B_ = 200.

The histograms of all *n*_C_ configurations were averaged. Finally, the histograms were multiplied by the factor *c*, 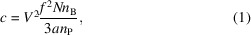
where *V* denotes the volume of a single particle. This ensures that, in the absence of interaction effects, the integral of the PDDF_i_s multiplied by 4π would equal *V*^2^.

### 2.2. Experiments

Lysozyme derived from chicken egg white was procured from Sigma–Aldrich (Source BCCL9074). Solutions were prepared in pure deionized water, as well as in 0.10 and 0.19 mol L^−1^ NaCl solutions, with target concentrations (weight/weight) of 1.0%, 2.5%, 5.0%, 10.0%, 15.0% and 20.0%. Subsequent references to samples will indicate their actual concentrations. All solutions were clear, with the exception of those at 14.9% and 20.0% in 0.19 mol L^−1^ NaCl, which were not investigated further.

The small-angle X-ray scattering patterns of the solutions were measured using an N8-Horizon (Bruker AXS, Germany). Each sample was measured between three and fifteen times, depending on contrast, with each measurement lasting 30 min. Sample transmissions were determined by measuring the samples and the empty beam with added glassy carbon for 10 s. Azimuthal integration yielded scattering curves of scattering intensities *I*(*q*) as a function of the magnitude of the scattering vector 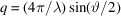, where λ is the wavelength of the radiation used and ϑ is the scattering angle. Repeated exposures were averaged, resulting in error bars for the intensities. The scattering curves of the appropriate solvent were subtracted from the sample curves after transmission correction. A constant background was estimated prior to subtraction using a Porod approximation to the high-*q* range of the scattering curves.

The scattering data were transformed into real space using the indirect Fourier transformation. Experimental scattering curves, approximations by IFT and the corresponding PDDF_i_s without further scaling are presented in Fig. 22 of the supporting information (SI). The PDDF_i_s were ultimately rescaled by dividing them by the value at *r* = 0.4 nm to facilitate the comparison of curves measured at different concentrations.

### 2.3. GIFT evaluation

The simulated PDDF_i_s were transformed into a scattering curve *I*(*q*) by numerical transformation:
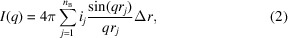
where *i*_*j*_ is the scaled height of the bin corresponding to a distance *r*_*j*_ and Δ*r* = *fa*/*n*_B_ is the width of a bin. This corresponds to Δ*r* = 0.082 nm for the simulated spheres, Δ*r* = 0.065 nm for oblate ellipsoids and Δ*r* = 0.051 nm for prolate ellipsoids. Error bars were calculated by propagating the uncertainties from the simulated pair distance distribution functions. Later in the text the PDDF_i_s are only shown up distances of 8 nm or less. Nevertheless, the full distance range was used for the calculation of *I*(*q*). All PDDF_i_s have reached zero within simulated uncertainties at this distance except for the *i*(*r*) of prolate ellipsoids at β = −1.5 *kT*.

GIFT evaluations were conducted in a stepwise manner. Initially, the averaged hard-sphere structure factor model (Brunner-Popela & Glatter, 1997 ▸), based on the Percus–Yevick solution (Percus & Yevick, 1958 ▸), was employed. If this approach failed to yield a PDDF_p_ without obvious distortions due to interaction effects, *i.e.* if the result indicated that the GIFT evaluation had failed, alternative structure factor models were utilized. Given that this situation arose only when the PDDF_i_ prior to GIFT exhibited a pronounced peak at large distances—typically indicative of attractive interactions—the GIFT evaluation using the square-well Percus–Yevick structure factor model (Innerlohinger *et al.*, 2004 ▸) was attempted next. Should this approach also fail to produce an acceptable PDDF_p_, the Teixeira model for fractal aggregates (Teixeira, 1988 ▸) was then applied.

## 3. Results and discussion

### 3.1. Effects due to excluded volume

**Figure 1:**
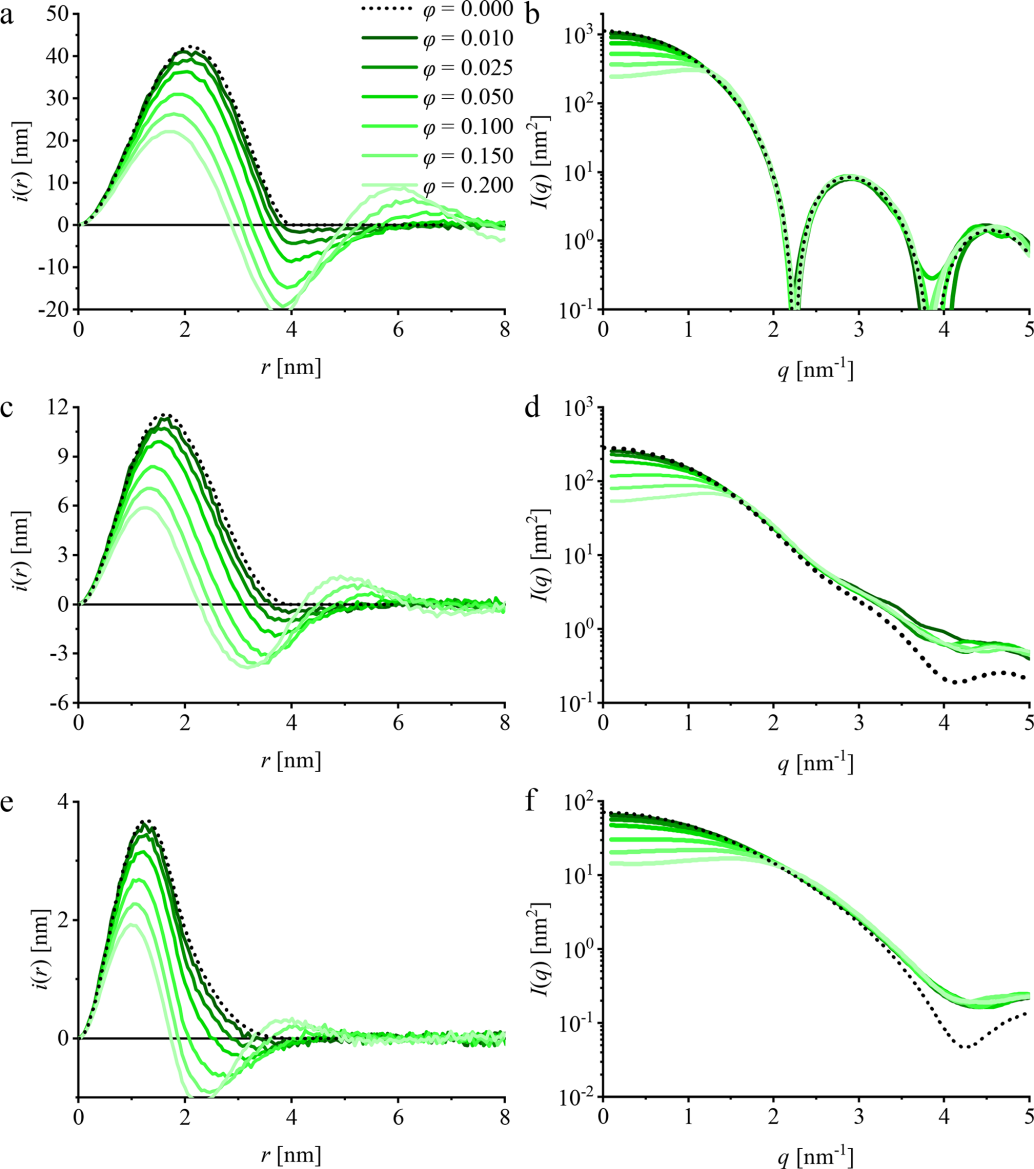
Pair distance distribution functions *i*(*r*) and scattering curves *I*(*q*) of particles at varying volume fractions, φ, interacting solely via excluded-volume effects. (*a*) and (*b*) Spheres with a radius of 2 nm; (*c*) and (*d*) oblate ellipsoids of revolution with half-axis lengths 1 and 2 nm; (*e*) and (*f*) prolate ellipsoids of revolution with half-axis lengths 1 and 2 nm. The dotted curves show the curves at infinite dilution.

Fig. 1 ▸(*a*) illustrates the well-established pair distance distribution functions of spheres, which interact exclusively through excluded-volume effects, as obtained by the Monte Carlo simulations. As the concentration increases, a pronounced minimum emerges near the diameter of the spheres, accompanied by a maximum at greater distances that shifts towards smaller values of *r* with rising volume fraction. These phenomena can be attributed to the addition of the system’s total correlation function, broadened by a cross term arising from the convolution of the electron density distributions of two proximate particles (Fritz-Popovski *et al.*, 2011 ▸). The reduction in the area under the PDDF_i_ curves with increasing volume fraction is mirrored in reciprocal space by a decrease in forward-scattering intensity [Fig. 1 ▸(*b*)], whereas the enhanced order gives rise to an interaction peak.

**Figure 2:**
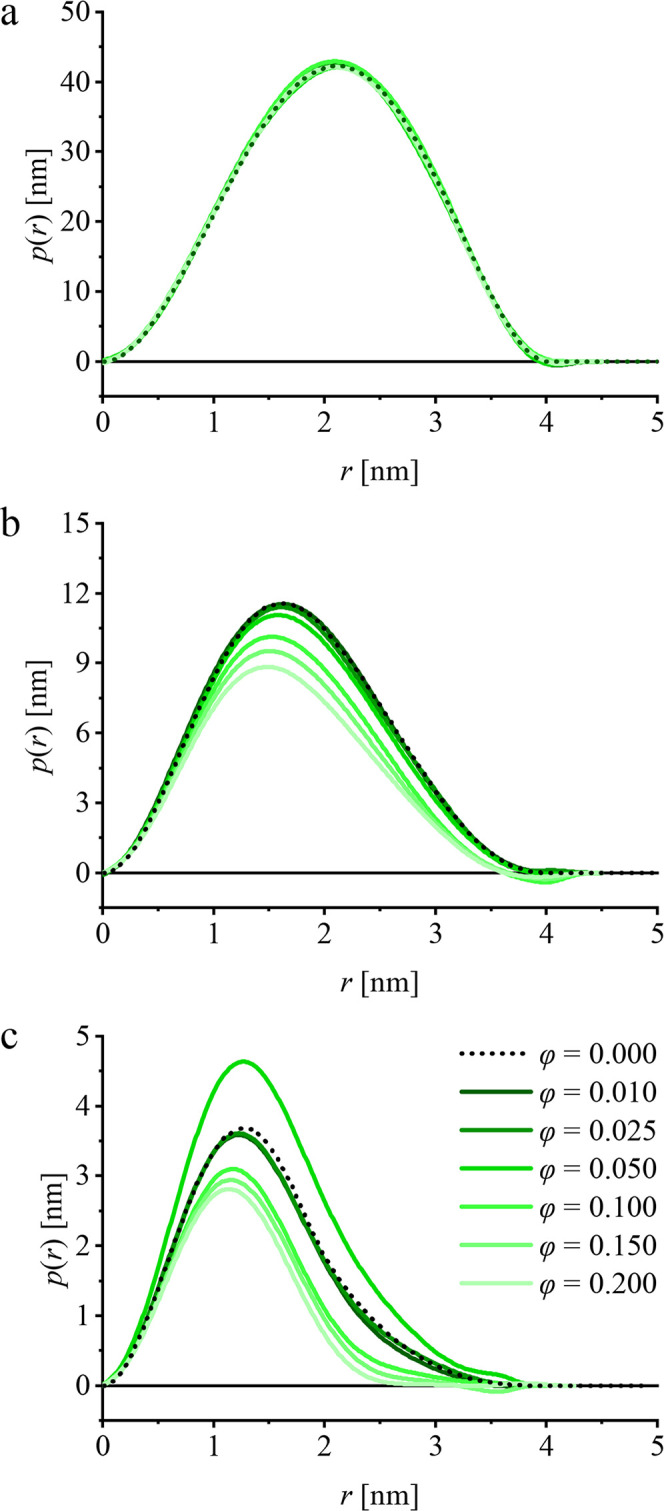
Pair distance distribution functions for the systems depicted in Fig. 1 ▸, incorporating interaction effects through an averaged hard-sphere structure factor, as determined using GIFT. (*a*) Spheres, (*b*) oblate ellipsoids and (*c*) prolate ellipsoids.

Oblate and prolate ellipsoids of revolution exhibit broadly similar behaviour [Figs. 1 ▸(*c*)–1 ▸(*f*)]. However, three notable distinctions can be observed: Firstly, the positions of the minima shift significantly more than in the case of spheres (SI, Fig. 1). Secondly, while the absolute shifts of the maxima are comparable, they occur at substantially smaller distances than the maximum dimension of the particles. Thirdly, the depths and heights of these extrema are less pronounced. Lastly, these effects are more pronounced for prolate ellipsoids than for oblate ones (SI, Fig. 2).

This behaviour can be elucidated through the theoretical framework governing the features of pair distance distribution functions for spheres. In essence, the minimum in the PDDF_i_ of interacting spheres arises from the excluded-volume effect, which gains prominence with increasing concentration. Conversely, the maximum is attributed to the next-neighbour peak. The significance of this contribution similarly grows with concentration, accompanied by an increase in the peak’s height.

Fundamentally, the same phenomena account for the minima and maxima in the simulated pair distance distribution functions of ellipsoids. However, the neighbouring peaks in the pair correlation functions are less pronounced (see SI, Fig. 5). Moreover, there is no longer a sharp step at the distance equal to the diameter of the sphere. Naturally, the centres of two neighbouring ellipsoids must be separated by at least the length of the shortest axis. However, depending on the mutual orientation of the axes, they could also overlap if their centres are separated by less than the length of the longest axes. Therefore, the total correlation function exhibits a gradual increase at distances ranging from the length of the shortest axis to the length of the longest axis. This increase becomes more pronounced with higher volume fractions. In the case of prolate ellipsoids, even the next-neighbour peak develops at these distances smaller than the maximum dimension of the particles.

This behaviour of the total correlation functions can elucidate the simulated pair distance distribution functions. The excluded-volume effect at small distances, up to the length of the shortest axis, is fundamentally similar to that observed for spheres. However, intra-particle contributions predominantly influence these distances in most cases. The onset of the increase in the total correlation function determines the position of the minimum. As this increase becomes more pronounced with higher concentrations, one observes a shift of the minimum to smaller distances with increasing volume fraction. Additionally, the minimum shifts to distances where intra-particle effects become increasingly significant, resulting in shallower minima. Similar considerations apply to the maxima. Importantly, the neighbouring peak is less pronounced for ellipsoids, leading to weaker maxima.

By employing the GIFT technique, one may aspire to eliminate interaction effects from the *i*(*r*) functions. This approach proves particularly effective for spherical particles, as illustrated in Fig. 2 ▸(*a*). This outcome should come as no surprise, given that the structure factor model utilized was the averaged hard-sphere model (Brunner-Popela & Glatter, 1997 ▸), which is founded upon the analytical Percus–Yevick result for hard spheres (Percus & Yevick, 1958 ▸). The parameters derived from the model align quite well with those simulated (Table 1 ▸). The sole exception is the estimated polydispersity at a volume fraction of 1%, which is hardly unexpected, considering the minimal interaction effects at such low concentrations.

The pair distance distribution functions derived by GIFT for oblate ellipsoids of revolution exhibit a shape remarkably similar to *p*(*r*) at infinite dilution [Fig. 2 ▸(*b*)]. Nevertheless, two observations are noteworthy: Commencing at a volume fraction of 5%, the height of the curve is underestimated. Additionally, starting at this concentration, the maximum of the calculated *p*(*r*) shifts slightly towards smaller distances with increasing concentration. This phenomenon exerts a quantitative influence on the interpretation of the function.

Qualitatively, one would still regard the particles as oblate ellipsoids, given that the shape of the *p*(*r*) curve has scarcely altered [SI, Fig. 6(*b*)]. Quantitatively, however, one would underestimate the length of the short axis. The values obtained for this parameter would decrease to as low as 0.89 nm for a volume fraction of 20%.

The findings suggest that the oblate elliptical shape exerts a negligible influence on long-range order at volume fractions up to 5%. The residual excluded-volume effect can be adequately described by an effective hard-sphere model. This description becomes less accurate at higher concentrations, as evidenced by an underestimation of the obtained volume fraction (Table 1 ▸). Consequently, this leads to an underestimation of the forward-scattering intensity, which is proportional to the area under the *p*(*r*) function. Ultimately, this manifests in the diminishing height of the pair distance distribution functions observed in Fig. 2 ▸(*b*).

The PDDF_p_s derived via GIFT for prolate ellipsoids at volume fractions of 1% and 2.5% exhibit a striking similarity to the theoretical curve [Fig. 2 ▸(*c*)]. The function obtained at a volume fraction of 5%, however, while possessing the expected shape, is notably elevated. At even higher concentrations, the heights decrease, akin to the behaviour observed in oblate ellipsoids. Nevertheless, the curve’s shape deviates significantly from the theoretical prediction, particularly within the distance range of 2 to 4 nm.

The parameters derived from the averaged hard-sphere model, when applied to data from prolate ellipsoids, reveal corresponding over- and underestimations of the volume fraction. The sample at 5% volume fraction also yields an unrealistically small interaction radius. During evaluation, the GIFT software identifies a local minimum that aligns more closely with solutions found at higher concentrations. Thus, one may infer that interactions at volume fractions below 5% are well described by the employed model. At 5%, the solution becomes unstable, whereas at higher concentrations, stable solutions are obtained which exhibit systematic deviations from the theoretical PDDF_p_.

Much like the case of oblate ellipsoids, the qualitative interpretation of the *p*(*r*) functions derived through GIFT for prolate ellipsoids remains feasible. However, at higher volume fractions, the length of the long axis is significantly underestimated (SI, Table 1). This discrepancy can be attributed to the challenge faced by the GIFT technique in distinguishing overlapping inter- and intra-particle effects within the range from the short axes’ length to that of the long axes. This issue is more pronounced for prolate particles than for oblate ones, as neighbouring particles are more likely to encroach within distances shorter than the ellipsoid’s maximum dimension. Consequently, two arbitrary points separated by such distances have a higher probability of belonging to different particles than in the case of oblate ellipsoids or spheres. It is, therefore, more challenging to entirely eliminate such effects using a model that serves as a rather imperfect approximation.

### 3.2. Attractive and repulsive contributions

**Figure 3:**
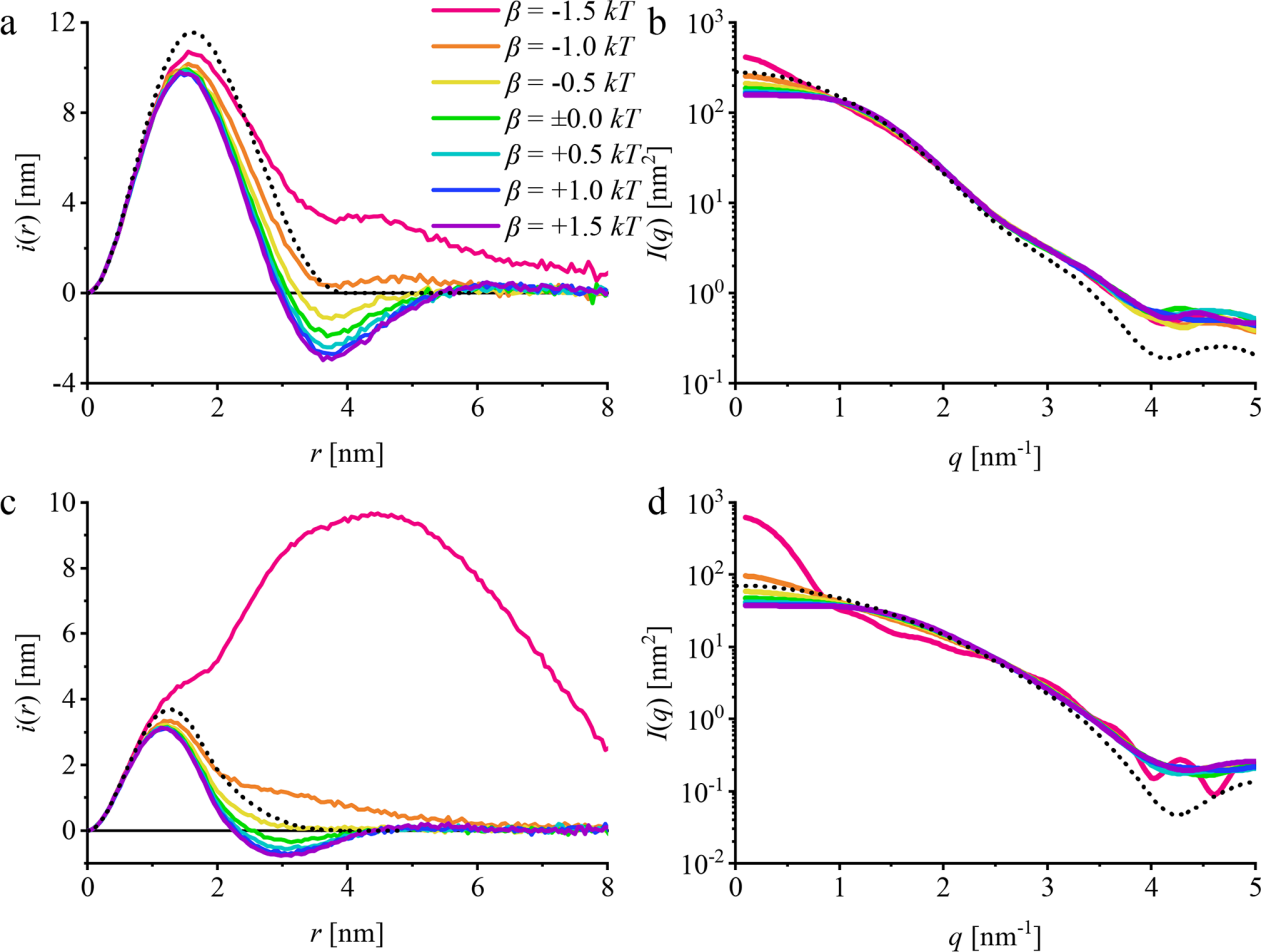
PDDF_i_*i*(*r*) and scattering functions *I*(*q*) of particles at a volume fraction φ = 0.05 interacting by different potentials. (*a*) and (*b*) oblate ellipsoids; (*c*) and (*d*) prolate ellipsoids.

Fig. 3 ▸ presents the PDDF_i_s and scattering curves *I*(*q*) of the oblate and prolate ellipsoids at a volume fraction of φ = 0.05, interacting via square-well and square-wall potentials.

The scattering curves show similar trends as β increases. The forward scattering decreases, consistent with increasing repulsion, while the onset of an interaction peak indicates higher long-range order. The curve for prolate ellipsoids at β = −1.5 *kT* [Fig. 3 ▸(*d*)] additionally shows many oscillations. This is due to the particles forming aggregated, large structures that can no longer be described correctly by the Monte Carlo simulation used. However, the results are reliable locally, which is also why *i*(*r*) is shown in Fig. 3 ▸(*c*) only up to 8 nm.

The PDDF_i_s in the presence of an additional repulsive interaction potential bear a resemblance to those derived for particles interacting solely through excluded-volume effects. This observation holds true for both oblate and prolate ellipsoids. The repulsive potential results in slightly more pronounced minima and maxima, although this effect is relatively minor. A shift in the position of these features is not observed.

This does not hold for attractive interaction potentials. Even a weak attraction, such as in the case of β = −0.5 *kT*, leads to a considerable reduction of the minimum. The effect is more pronounced for prolate particles, where the minimum has essentially vanished, than for oblate ones, where a weak minimum still prevails. Stronger attraction leads to the formation of a broad peak at large distances.

Particularly, the prolate particles at β = −0.5 *kT* highlight a potential issue when dealing with such systems. The absence of any minimum that would indicate repulsive interactions, as well as the lack of a tail at large distances that would signify the onset of aggregate formation, leaves no visible evidence of interaction effects. Consequently, one might interpret the PDDF_i_ as representing non-interacting particles unless additional information were available. Such information could include knowledge of the actual particle concentration, which would raise concerns as, at a volume fraction of 5%, one would expect to observe interaction effects in the *i*(*r*) profile.

**Figure 4:**
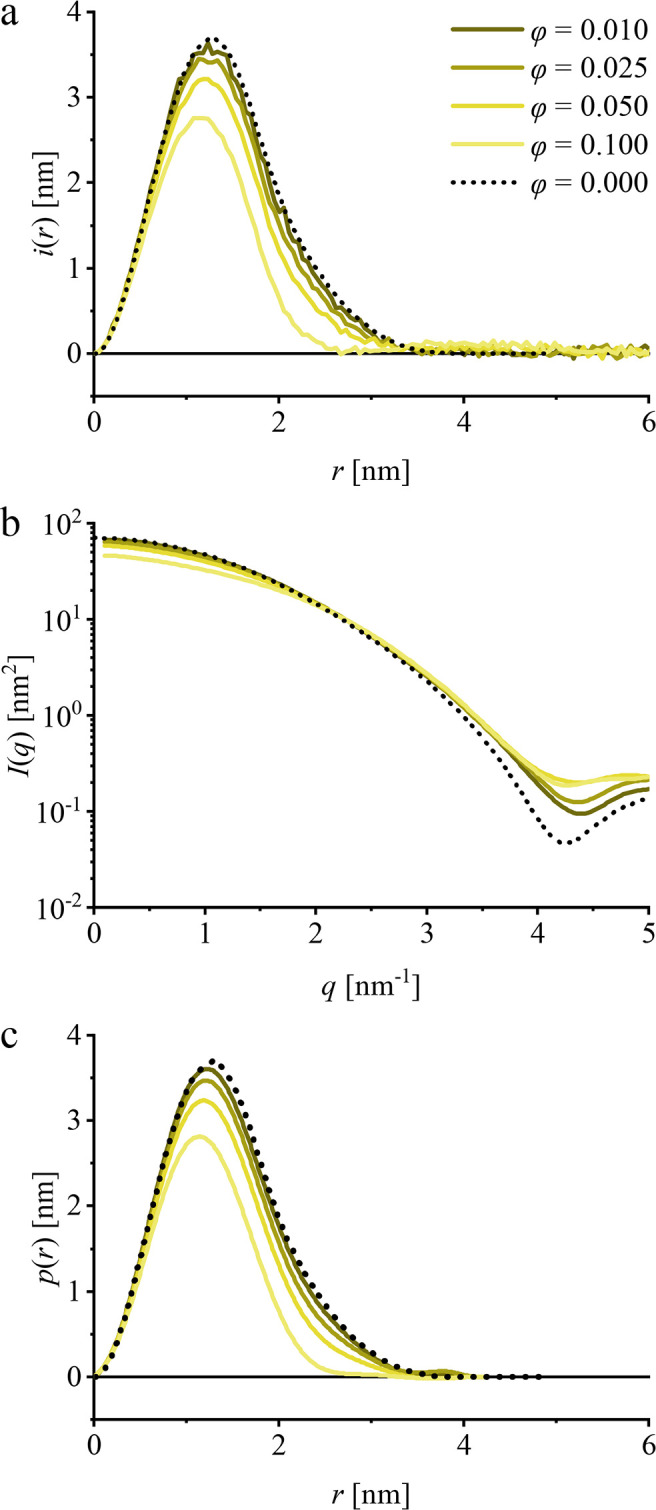
(*a*) Simulated PDDF_i_s of prolate particles interacting with a square-well potential at β = −0.5 *kT*. (*b*) Corresponding scattering curves. (*c*) Results of GIFT evaluation.

Upon examining this behaviour for different volume fractions at β = −0.5 *kT*, one finds that interaction effects are practically imperceptible up to the maximum studied. Fig. 4 ▸ illustrates that the *i*(*r*) function merely becomes less asymmetric. As the volume fraction increases, the scattering curves broaden and the forward intensity decreases, an effect that resembles a decrease in particle size. A GIFT evaluation of the corresponding scattering data does not yield *p*(*r*) functions that deviate significantly from the simulated *i*(*r*) functions. This observation is supported by model approximations, which result in an increasingly more global shape for higher concentrations (SI, Table 10).

**Figure 5:**
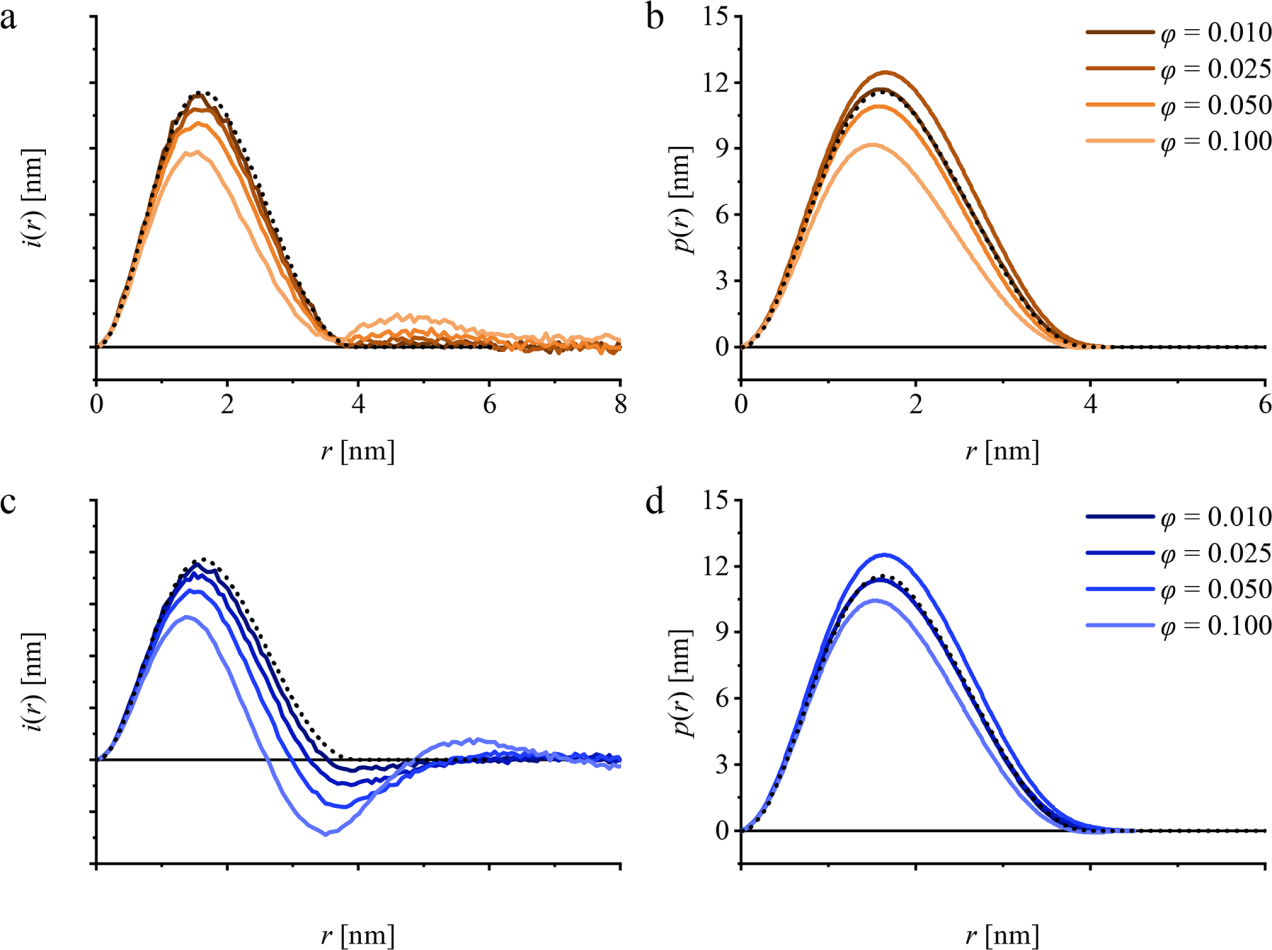
Pair distance distribution functions of oblate particles interacting with a square-well potential at (*a*) and (*b*) β = −1.0 *kT*; (*c*) and (*d*) β = +1.0 *kT*. (*a*) and (*c*) simulated *p*(*r*) functions; (*b*) and (*d*) those obtained by GIFT.

Fig. 5 ▸ illustrates the *i*(*r*) functions of oblate particles at β = ±1.0 *kT*, along with the corresponding *p*(*r*) functions obtained from the GIFT evaluations. The simulated curves for particles exhibiting an attractive interaction potential reveal the formation of a weak peak at large distances. Conversely, repulsive interactions yield *i*(*r*) functions that closely resemble those observed for β = 0, characterized by more pronounced minima and maxima at large distances. The GIFT evaluation performs qualitatively well, producing *p*(*r*) functions that correspond to the correct particle shape, albeit with varying scaling of the height. This variability is also evident in the fitted parameters for attractive particles, which exhibit no clear trend (Table 2 ▸). In the presence of repulsive interactions, the GIFT evaluations demonstrate at least a reasonable trend in terms of volume fraction. However, the averaged hard-sphere model also encounters difficulties in fully describing the situation, as reflected in the variations of the radius and polydispersity

**Figure 6:**
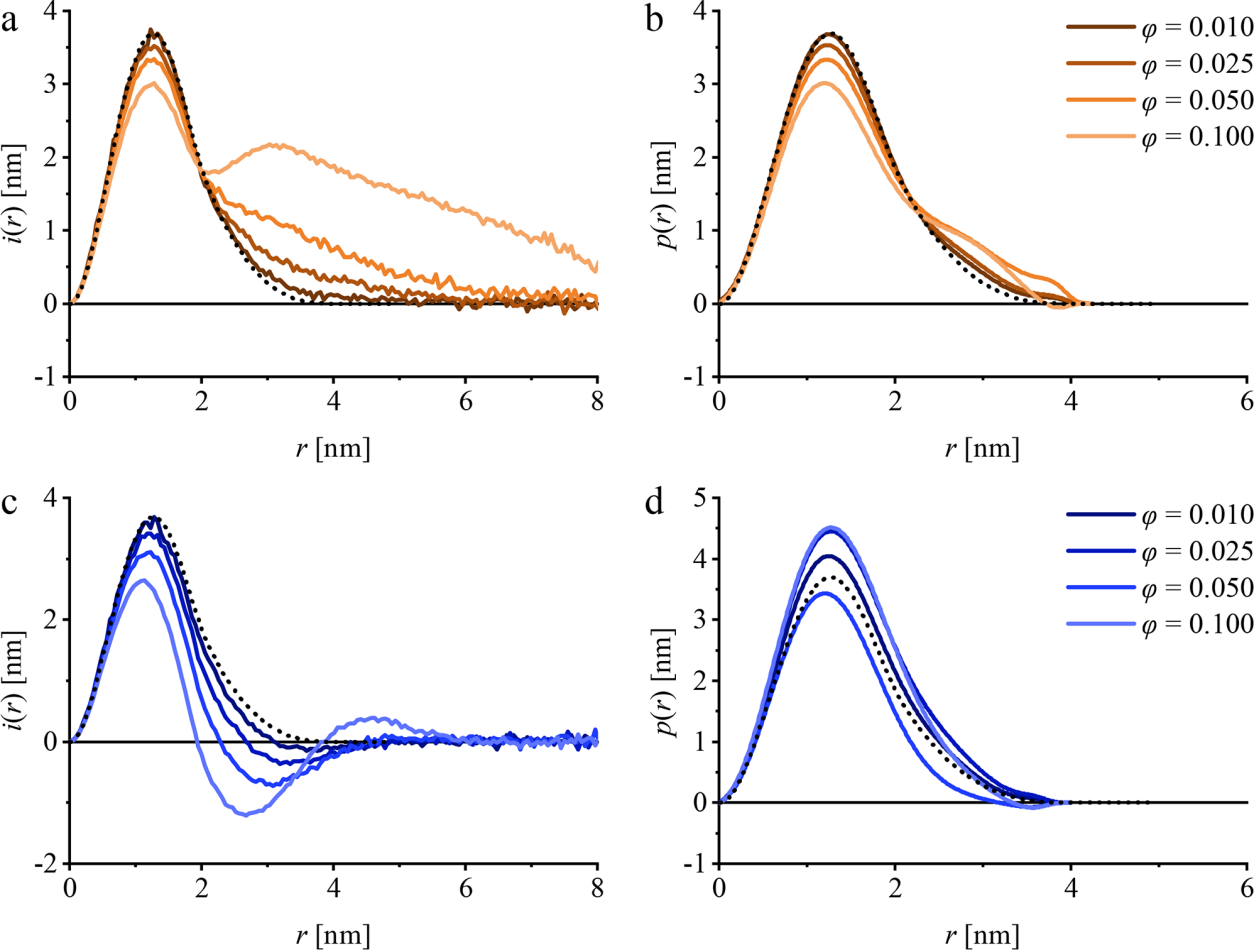
Pair distance distribution functions of prolate particles interacting with a square-well potential at (*a*) and (*b*) β = −1.0 *kT*; (*c*) and (*d*) β = +1.0 *kT*. (*a*) and (*c*) simulated *p*(*r*) functions; (*b*) and (*d*) those obtained by GIFT.

The situation for prolate ellipsoids exhibits similar characteristics. In this case as well, the peak at large distances, indicative of attractive interactions, is evident in the PDDF_i_s. Furthermore, the functions closely resemble those demonstrating simple excluded-volume effects for repulsive interactions (Fig. 6 ▸). The GIFT evaluations also yield PDDF_p_s that are qualitatively consistent with those of non-interacting particles. As observed with oblate particles, the fitted parameters obtained through GIFT highlight the limitations of the technique when applied to such particles (Table 2 ▸). The primary distinction lies in the consistently high polydispersity values obtained for the repulsive particles.

### 3.3. Comparison with experiments

Crystallographic data (Sauter *et al.*, 2001 ▸) were used to compute the small-angle scattering curve of lysozyme in order to estimate the ellipsoid that describes the protein in the absence of interaction. An ellipsoid of revolution with a single axis of 2.62 nm and double axes of 1.54 nm was found to be the best model for these data (see section *Approximation of Lysozyme Structure by Ellipsoid* in the supporting information).

**Figure 7:**
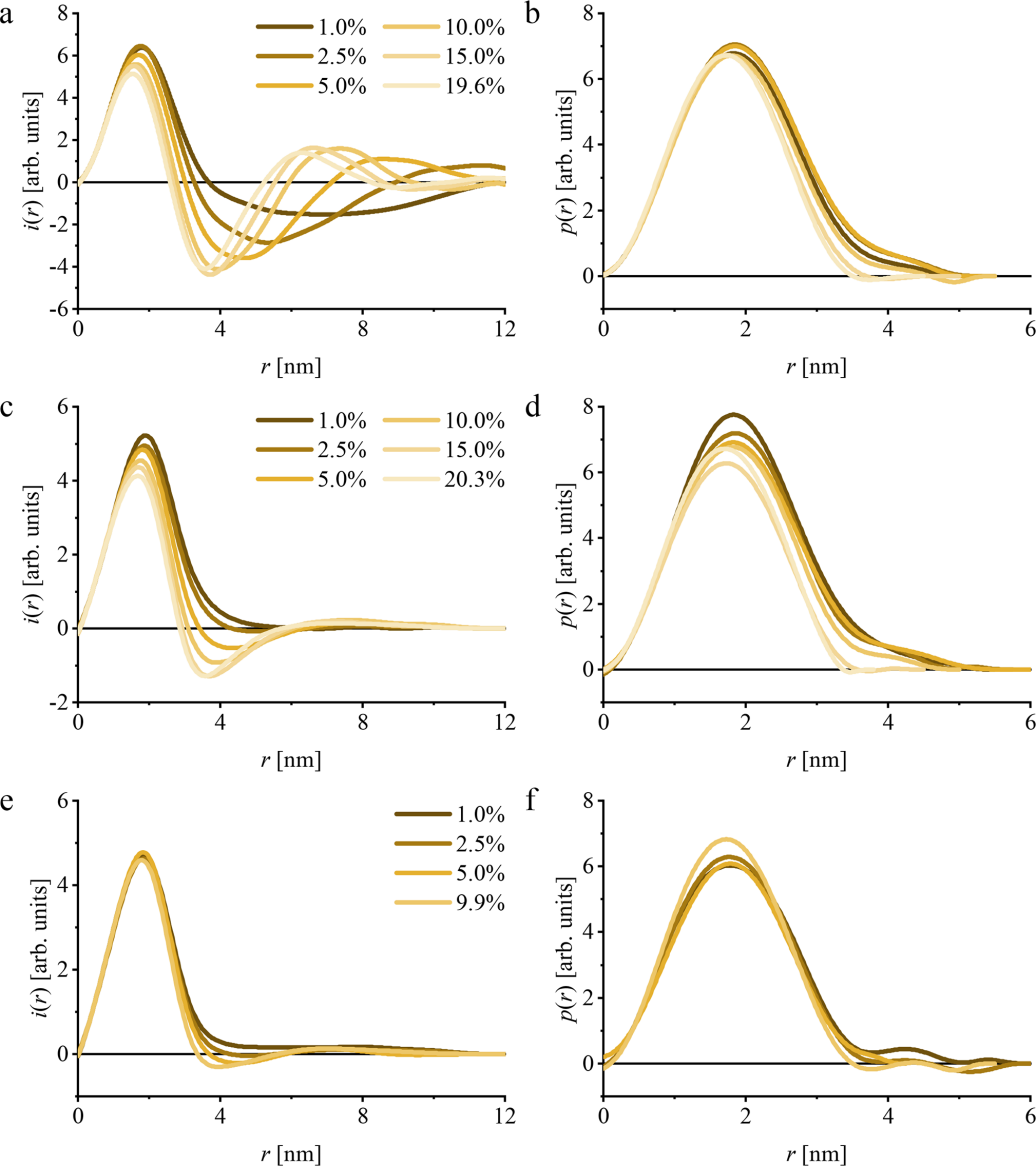
Pair distance distribution functions of lysozyme at different concentrations in (*a*) deionized water, (*c*) 0.10 mol L^−1^ aqueous NaCl and (*e*) 0.19 mol L^−1^ NaCl solutions. (*b*), (*d*) and (*f*) show the corresponding GIFT results.

The charge of the proteins leads to repulsive interactions in addition to excluded-volume effects. Therefore, one expects to find a situation similar to that presented in Fig. 6 ▸(*c*). The actual *i*(*r*) functions of lysozyme in aqueous solution as obtained from the scattering curves shown in SI Fig. 22 are shown in Fig. 7 ▸(*a*). The agreement is good if one takes into account that the axis ratio of the simulated ellipsoid differs considerably from that found for lysozyme. Both the minima and the maxima shift to smaller distances with increasing concentration, while they become more pronounced. The position of the main minimum is like in Fig. 6 ▸(*c*) or Fig. 1 ▸(*c*) slightly above twice the length of the short axes at high concentrations.

Adding 0.1 mol L^−1^ of salt leads to electrostatic screening, while the van der Waals attraction is still present. The resulting DLVO potential is therefore less repulsive, which leads to weaker minima and maxima as seen in Fig. 7 ▸(*c*).

An elevation of salt concentration to 0.19 mol L^−1^ yields pair distance distribution functions [Fig. 7 ▸(*e*)] that bear resemblance to those illustrated in Fig. 4 ▸. The scattering results may also be characterized by ellipsoids of revolution devoid of interaction effects, although a minor negative component is observed at concentrations of 5% and 9.9%, potentially indicating an actual non-negligible concentration. The corresponding model parameters are given in SI Table 11.

The results of a GIFT analysis of the lysozyme scattering curves are shown in Figs. 7 ▸(*b*), 7 ▸(*d*) and 7 ▸(*f*). At low salt and lysozyme concentrations, they show good agreement with the simulated prolate ellipsoids in Fig. 6 ▸. At higher protein concentrations, the *p*(*r*) functions more closely resemble those of spheres (see also SI, Table 15), consistent with the trends observed for interacting prolate ellipsoids.

## 4. Conclusions

The presence of interaction effects renders the interpretation of the pair distance distribution function obtained from small-angle scattering experiments on ellipsoids of revolution more challenging than in the case of globular particles.

Firstly, rules of thumb that link the position of a minimum in the PDDF_i_ to the diameter of a hard-sphere dispersion cannot be applied to ellipsoids. In such cases, the position shifts with increasing concentration from twice the length of the long axis towards twice the length of the short axis. However, the actual minimum diameter of the ellipsoids was not attained within the concentration range studied.

Secondly, employing the GIFT technique in combination with the averaged hard-sphere structure factor model yields PDDF_p_s that qualitatively agree with the actual ones of the particles in most instances. High concentrations of oblate ellipsoids might lead to a slight underestimation of the length of the short axis. High concentrations of prolate ellipsoids can still be recognized as elongated particles, and their short axes can be estimated reasonably well. Their actual ellipsoidal nature might, however, be difficult to discern.

Thirdly, additional repulsive interactions do not greatly alter the situation compared with excluded-volume effects alone. A GIFT evaluation using the averaged hard-sphere structure factor model provides satisfactory results in terms of PDDF_p_s.

Fourthly, attractive interactions can generally be treated effectively using appropriate structure factor models developed for spherical particles. However, situations may arise where the PDDF_i_ obtained by IFT is nearly indistinguishable from that of spheres without interaction effects. A GIFT analysis will fail in these cases. Nevertheless, the PDDF_p_s obtained by GIFT in nearly all other cases are rather similar to those of the dilute particles, leading to a correct determination of the particle size and shape.

One general conclusion may be drawn from these findings on GIFT evaluations of such scattering data. The *i*(*r*) functions of strongly interacting ellipsoids obtained by means of IFT can be difficult to interpret, whereas the *p*(*r*) functions computed by GIFT are only qualitatively accurate. From these, one may infer the ellipsoidal shape of the particles and obtain rough estimates of the axis lengths. However, such results should be regarded as provisional. As a next step, more specific models (Hansen, 2013 ▸), informed by the inferred ellipsoidal geometry, should be employed to derive reliable particle parameters.

The introduction of structure factor models for ellipsoidal particles into the GIFT technique might improve the situation and allow for this next step to be performed within the framework of the GIFT software. The situation would be similar to that of charged particles, where also two GIFT evaluation steps are required to obtain results that can be interpreted fully (Fritz *et al.*, 2000 ▸). However, this requires the incorporation of effective structure factor models for ellipsoids, like those based on the decoupling approximation, the local monodisperse approximation or the approximation introduced by Hansen (2013 ▸).

## 5. Related literature

The following references are cited only in the supporting information: Glatter (1980 ▸).

## Supplementary Material

Data that have been simulated but are not discussed in detail within the paper. Additionally the approximation of the protein structure from crystallography by an ellipsoid, and the scattering curves of the experimental data with fits as well as fitting parameters. Finally the scattering curves obtained by transformation of the simulated PDDFs and their approximation using GIFT. DOI: 10.1107/S1600576726004383/jl5130sup1.pdf

## Figures and Tables

**Table 1:** Parameters obtained by GIFT of the systems shown in Fig. 1 ▸ for the structure factor models of hard-sphere particles φ: volume fraction; *r*: radius; and σ: relative standard deviation of size distribution.

	Sphere			Oblate			Prolate		
φ	φ	*r*	σ	φ	*r*	σ	φ	*r*	σ
[%]	[%]	[nm]	[ ]	[%]	[nm]	[ ]	[%]	[nm]	[ ]
1.0	0.9	1.89	0.40	1.2	1.73	0.40	0.4	1.91	0.00
2.5	2.5	1.97	0.18	2.5	1.71	0.00	2.4	1.47	0.00
5.0	4.8	2.01	0.06	4.7	1.74	0.00	8.7	1.00	0.56
10.0	10.1	1.96	0.09	8.5	1.74	0.00	7.1	1.45	0.12
15.0	14.7	1.99	0.00	12.9	1.71	0.00	10.9	1.31	0.13
20.0	19.5	1.98	0.00	17.0	1.68	0.03	14.7	1.24	0.18

**Table 2:** Parameters obtained by GIFT for the structure factor of attractive and repulsive ellipsoids Attractive (square-well Percus–Yevick model): φ′: volume fraction; *r*: radius; *w*/*r*: relative well width; β/*kT*: well depth. Repulsive (averaged hard-sphere model): φ′: volume fraction; *r*: radius; σ: relative standard deviation of size distribution.

		β = −1.0 *kT*				β = +1.0 *kT*		
	φ	φ′	*r*	*w*	β/*kT*	φ′	*r*	σ
	[%]	[%]	[nm]	[ ]	[ ]	[%]	[nm]	[ ]
Oblate	1.0	0.8	1.23	0.99	−0.07	1.2	1.83	0.00
	2.5	4.4	1.46	0.20	−0.59	3.4	1.84	0.00
	5.0	1.9	1.29	0.91	−0.14	8.4	1.71	0.26
	10.0	2.6	1.89	0.24	−1.00	12.1	1.86	0.07
Prolate	1.0	0.3	1.32	0.80	−0.31	2.8	1.01	0.69
	2.5	0.3	1.22	0.79	−0.80	6.9	1.13	0.55
	5.0	0.2	1.20	0.91	−1.19	5.9	1.60	0.19
	10.0	1.4	1.11	0.89	−0.73	17.1	1.30	0.32
