# Supplementary material for: Analysis of small-angle scattering data of interacting ellipsoids using pair distance distribution functions
Source: J Appl Crystallogr. 2026 Jun 15;59(Pt 4):1025–35. doi: 10.1107/S1600576726004383 (PMC13440061; doi:10.1107/S1600576726004383)
Supplement: Supplementary file 1 [file j-59-01025-sup1.pdf]

JOURNAL OF
APPLIED
CRYSTALLOGRAPHY

Volume 59 (2026)

Supporting information for article:

Analysis of small-angle scattering data of interacting ellipsoids using pair distance distribution functions

Gerhard Fritz-Popovski

Pair Distance Distribution Functions of Interacting Ellipsoids

Supplementary Information

Gerhard Fritz-Popovski

1 Positions of Minima and Maxima in $p(r)$ Functions

The pair distance distribution functions for ellipsoids interacting solely through excluded volume effects have been computed as detailed in the main text. The positions of the minima and maxima within these $p(r)$ functions shift with increasing volume fraction (refer to Figure 1 in the main text). Figure SI-1 illustrates these positions for particles interacting exclusively via excluded volume effects.

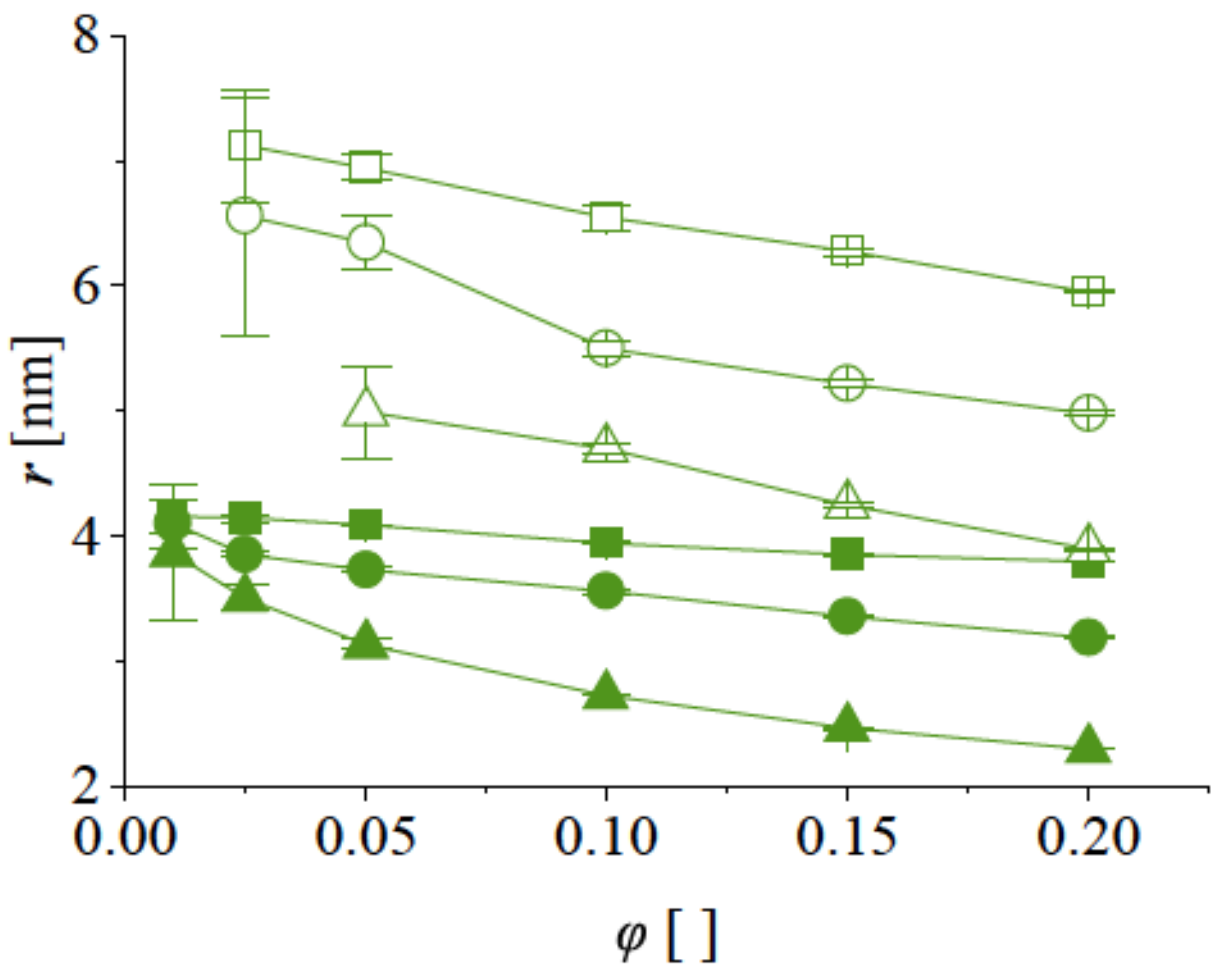

Figure SI–1 : Distances, where minima (solid) and maxima (open) occur due to interactions. Squares: spheres, circles: oblate ellipsoids, triangles: prolate ellipsoids.

Figure SI-2 shows the depths and heights of these minima and maxima. The values are plotted relative to the maximum height of the pair distance distribution function at infinite dilution.

Figures SI-3 and SI-4 show the positions of the minima and maxima in the $p(r)$ function of oblate and prolate ellipsoids that interact by square well and square wall potentials.

2 Pair Correlation Functions

Only the pair correlation functions for systems interacting exclusively through excluded volume effects were computed.

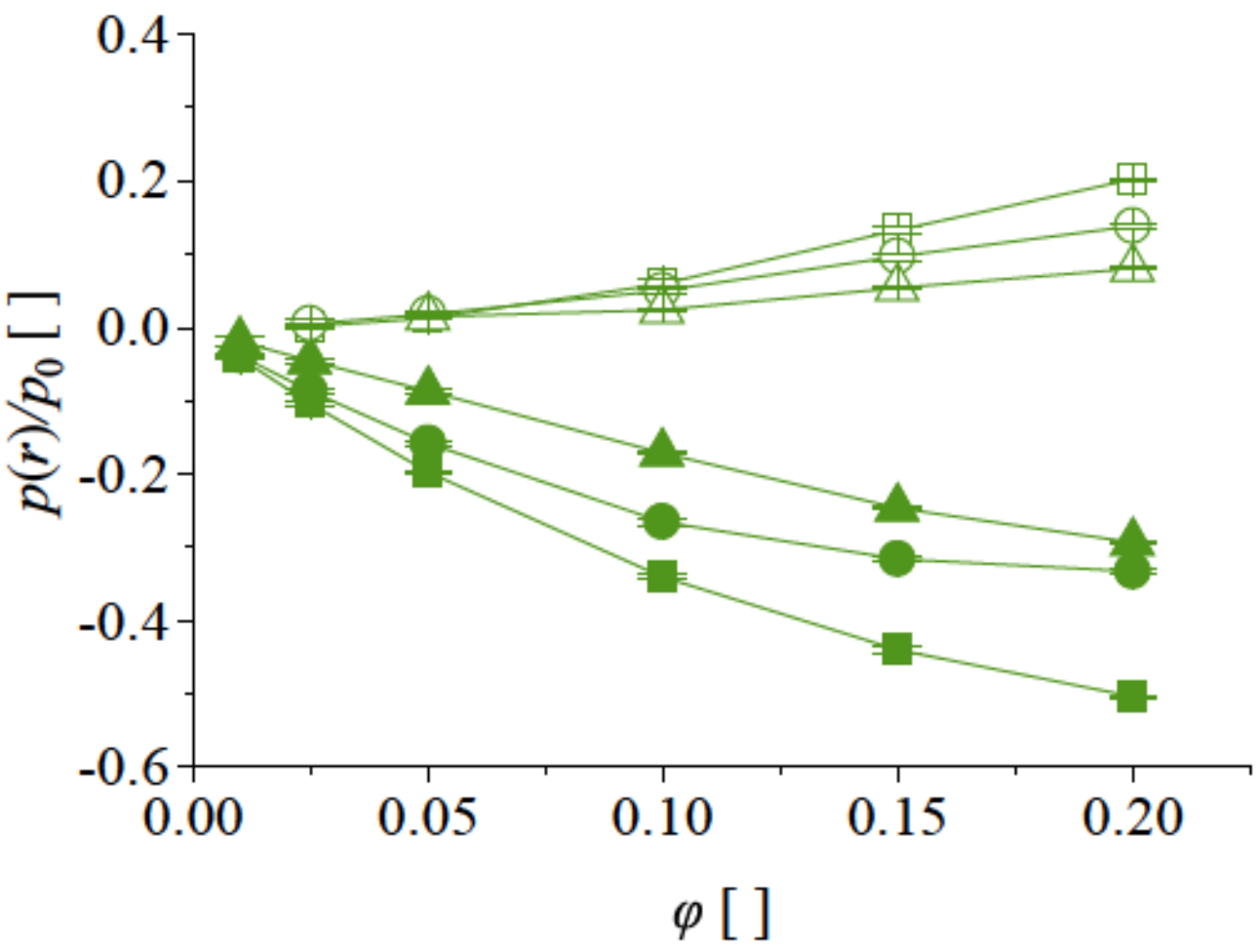

Figure SI–2 : Relative depth and heights of minima (solid) and maxima (open) that occur due to interactions. Squares: spheres, circles: oblate ellipsoids, triangles: prolate ellipsoids.

The pair correlation functions $g(r)$ (Fig. SI-5) were determined using essentially the same software that simulated the pair distance distribution functions $p(r)$. However, while $p(r)$ was probed using 10^6 pairs of points within the simulated box, each configuration provided $600\varphi \cdot (600\varphi - 1)/2$ pairs of particle centres. Consequently, to maintain approximately similar statistical uncertainties for all volume fractions, $36/\varphi^2$ equilibrated configurations were simulated, resulting in 5.04×10^6 to 6.43×10^6 pairs of points. These were taken into account for the determination of the $g(r)$ function.

The $g(r)$ function was approximated by the number of particle centres within a spherical shell centred at the centre of a given particle, with an inner radius r and an outer radius $r + \Delta r$, divided by the number of particle centres expected within such a shell if the particles were distributed completely randomly.

The total correlation function $h(r)$, mentioned in the main text, is related to the pair correlation function by $g(r) = h(r) + 1$. Therefore, $h(r)$ is negative at small distances, which facilitates the explanation of the negative parts of $p(r)$, while $g(r)$ is more straightforward to discuss in terms of probabilities.

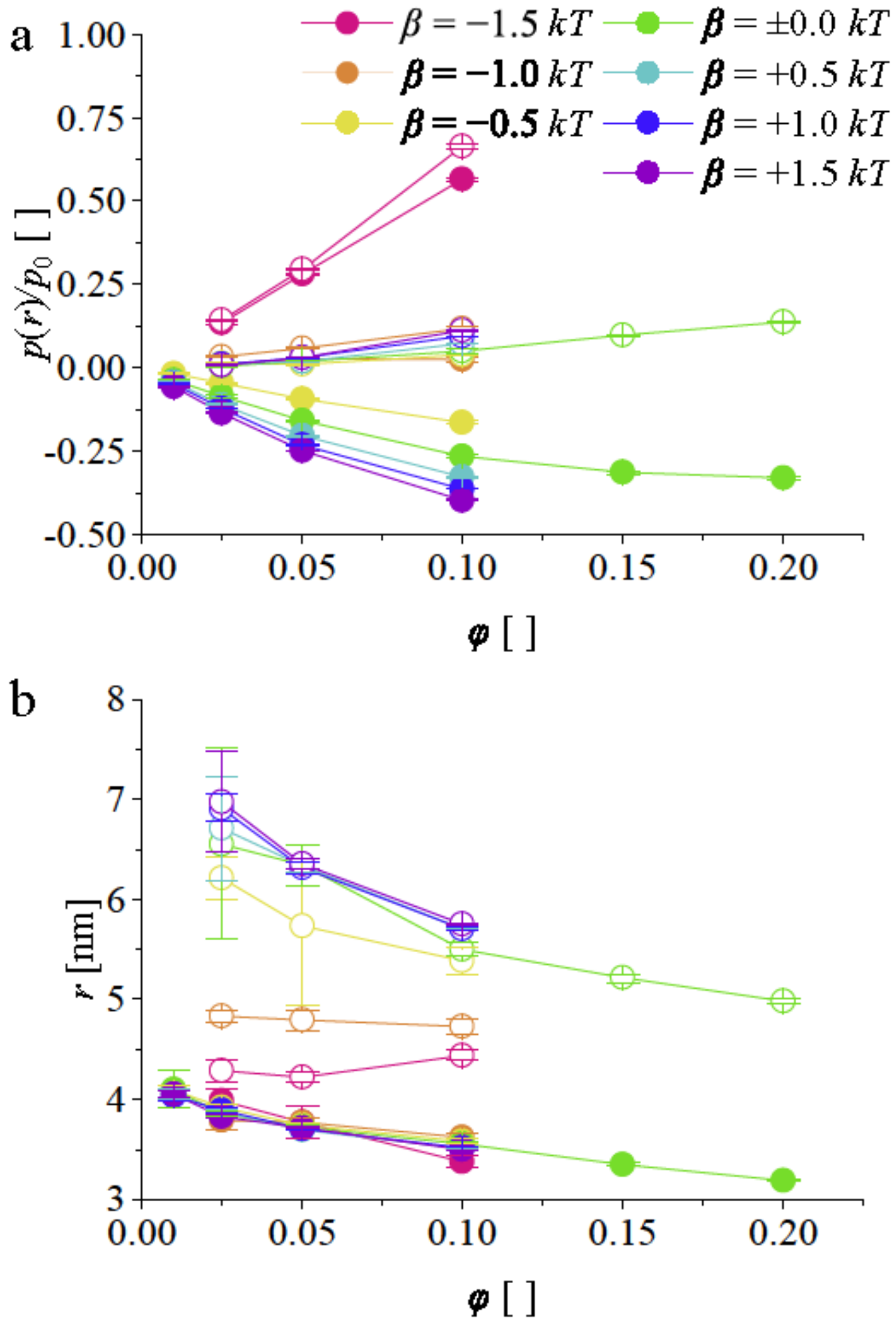

Figure SI–3 : Relative heights (a) and positions (b) of the minima (solid symbols) and maxima (open symbols) in the $p(r)$ functions of oblate ellipsoids.

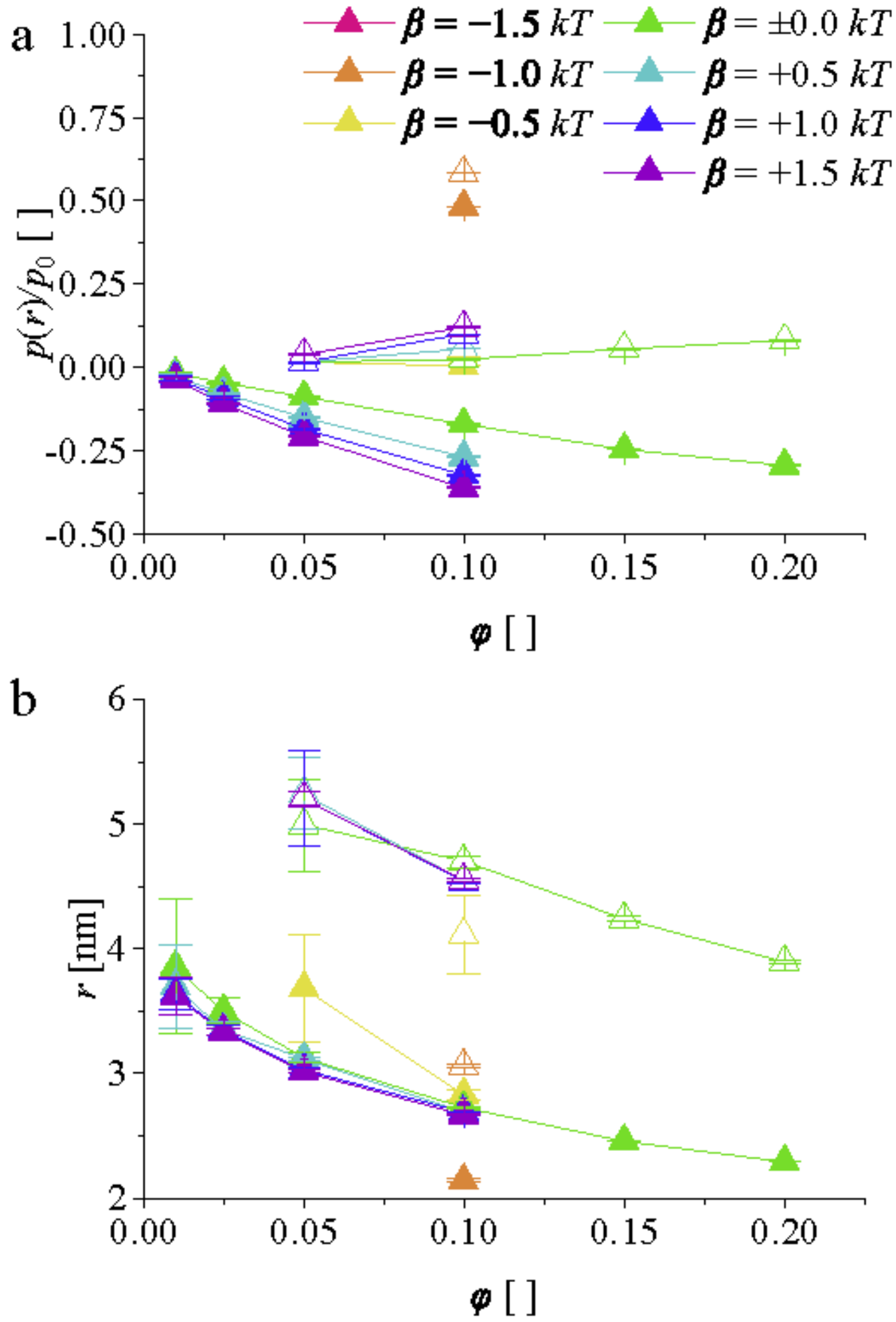

Figure SI–4 : Relative heights (a) and positions (b) of the minima (solid symbols) and maxima (open symbols) in the $p(r)$ functions of prolate ellipsoids.

3 GIFT Results Scaled to Same Height

The interpretation of the pair distance distribution functions in terms of particles is primarily dependent upon their shape, whereas the height is determined by contrast and concentration. To differentiate between various particle shapes, it may thus be beneficial to normalise the $p(r)$. Figure SI-6 presents the $p(r)$ functions derived from GIFT, scaled to the same maximum value. The curves correspond to those illustrated in Figure 2 of the main text.

4 Fit of Ellipsoids to GIFT Results

The interpretation of the $p(r)$ functions derived from a GIFT evaluation can be accomplished through a two-step process. The initial step involves the qualitative interpretation of the function's shape as that of an ellipsoid. The subsequent step entails approximating the corresponding theoretical model. This was achieved by applying the theoretical scattering curve of an ellipsoid of revolution [1] to the form factor, which serves as the reciprocal space analogue of the $p(r)$ function.

Table SI-1 presents the resulting fit parameters. The contrast relative to the theoretical value and the lengths of the short axes were determined well in most instances. However, the lengths of the long axes were systematically underestimated at higher volume fractions.

5 PDDFs of Ellipsoids with Attractive and Repulsive Interaction

5.1 Oblate Ellipsoids with $\beta = -1.5\ kT$

Figure SI-7a shows the simulated $p(r)$ functions of oblate ellipsoids interacting with an attractive potential having a maximum effect of $\beta = -1.5\ kT$. Figure SI-7b shows the corresponding results from a GIFT evaluation.

The GIFT evaluation was based on the SMSA closure relation for the structure factor, since a maximum is seen at large distances. The structure factor parameters are given in table SI-2. The basic shape of the $p(r)$ function is obtained well, although its height is underestimated in most cases. Nevertheless, one would interpret the size and shape of the particles correctly.

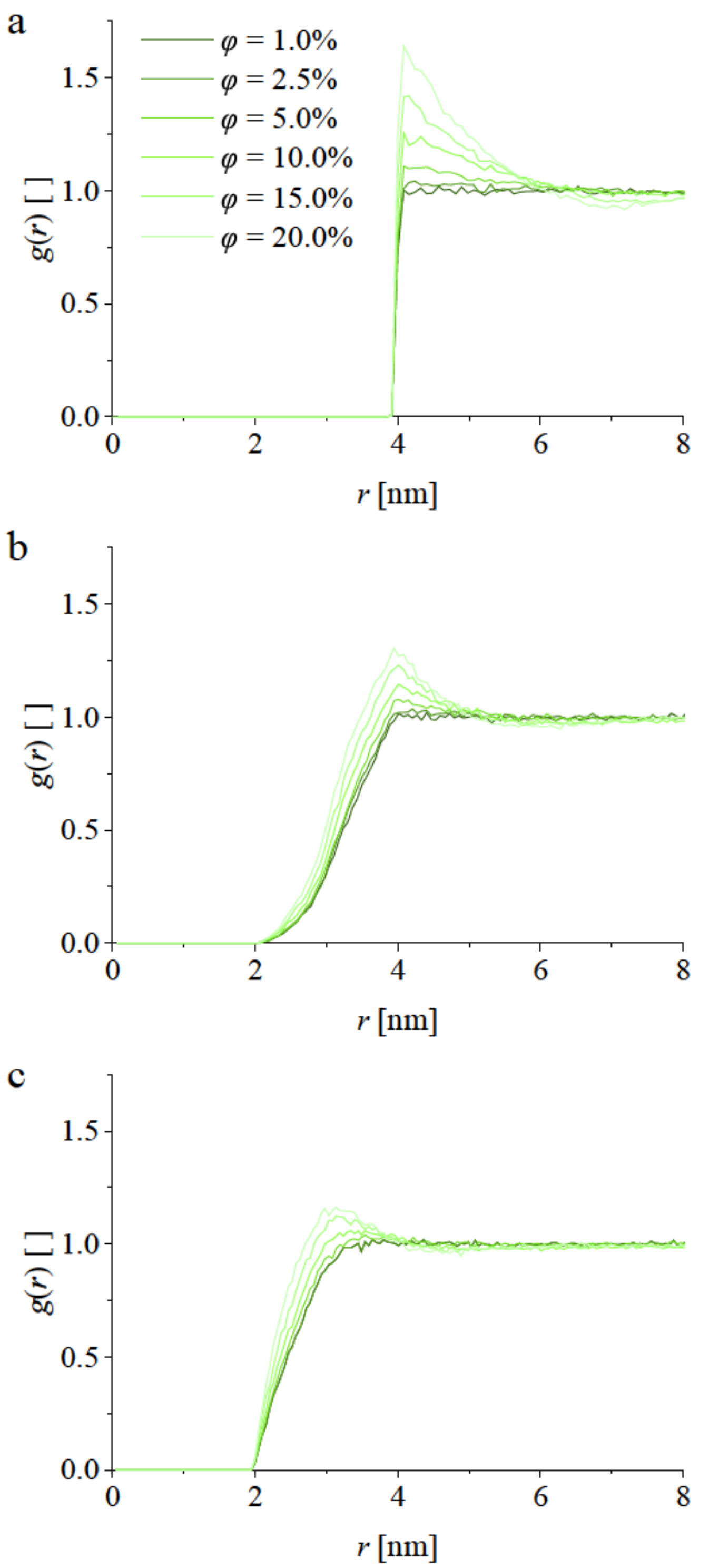

Figure SI–5 : Pair correlation functions $g(r)$ of spheres (a), oblate ellipsoids (b), and prolate ellipsoids (c).

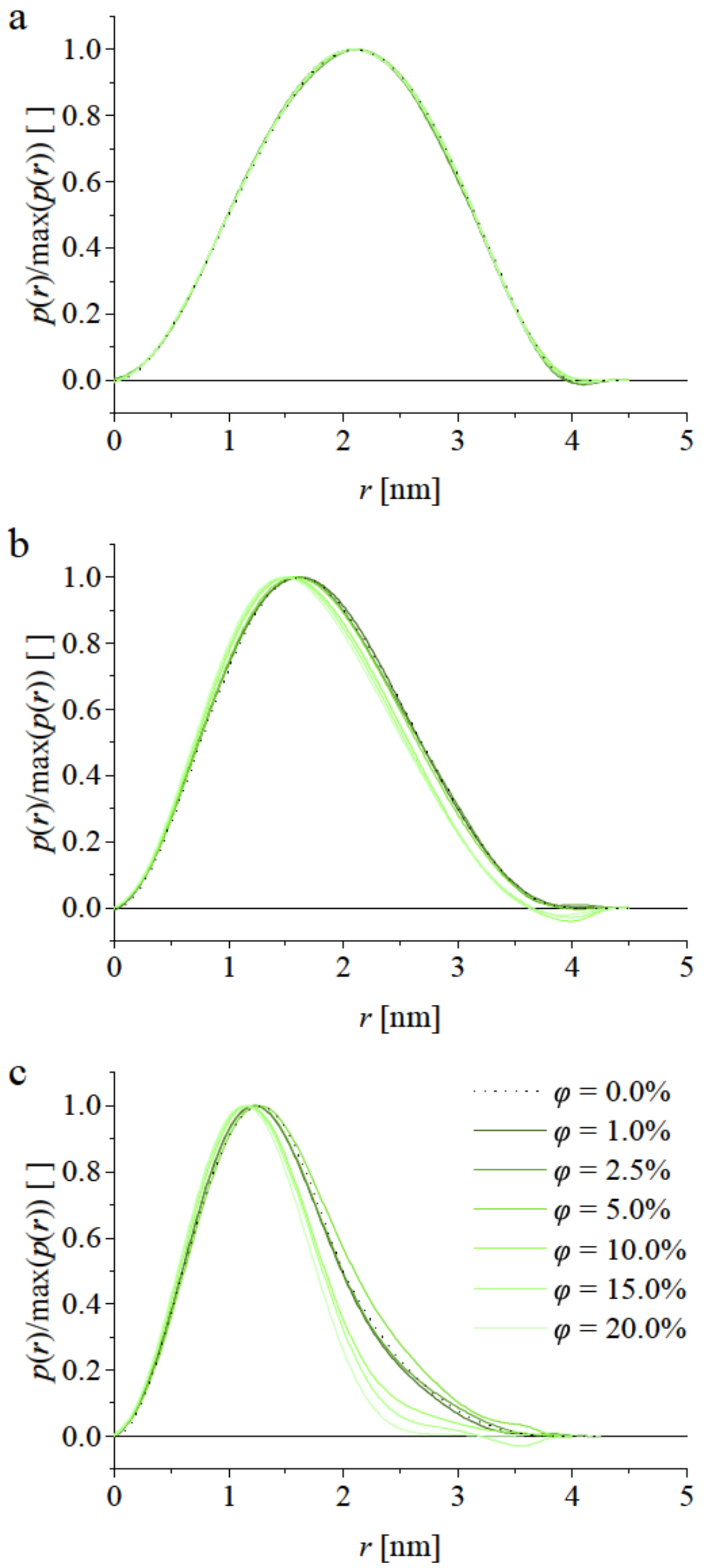

Figure SI–6 : Pair distance distribution functions as obtained by GIFT of spheres (a), oblate ellipsoids (b), and prolate ellipsoids (c). The functions are scaled to same maximum height.

5.2 *Oblate Ellipsoids with* $\beta = -0.5\ kT$

The $p(r)$ function of oblate particles at $\beta = -0.5\ kT$ are shown in figure SI-8a. They only show minima close to the particle diameter. Therefore, the GIFT analysis was based on the averaged PY hard-sphere structure factor model. The resulting $p(r)$ functions are shown in fig-

Table SI–1 : Parameters derived through the approximation of an ellipsoid of revolution to the form factor obtained from the GIFT calculation. φ: Simulated volume fraction, a: single half-axis, b: double half-axis, $\Delta\rho/\Delta\rho_0$: contrast

φ	Oblate			Prolate		
	a	b	$\Delta\rho/\Delta\rho_0$	a	b	$\Delta\rho/\Delta\rho_0$
[%]	[nm]	[nm]	[ ]	[nm]	[nm]	[ ]
1.0	0.98	2.01	1.01	1.95	1.00	1.00
2.5	0.99	2.00	1.00	2.16	1.01	0.93
5.0	0.98	1.98	1.01	2.13	1.03	1.02
10.0	0.95	1.92	1.04	1.71	0.97	1.07
15.0	0.92	1.92	1.04	1.62	0.97	1.09
20.0	0.89	1.90	1.05	1.49	0.97	1.12

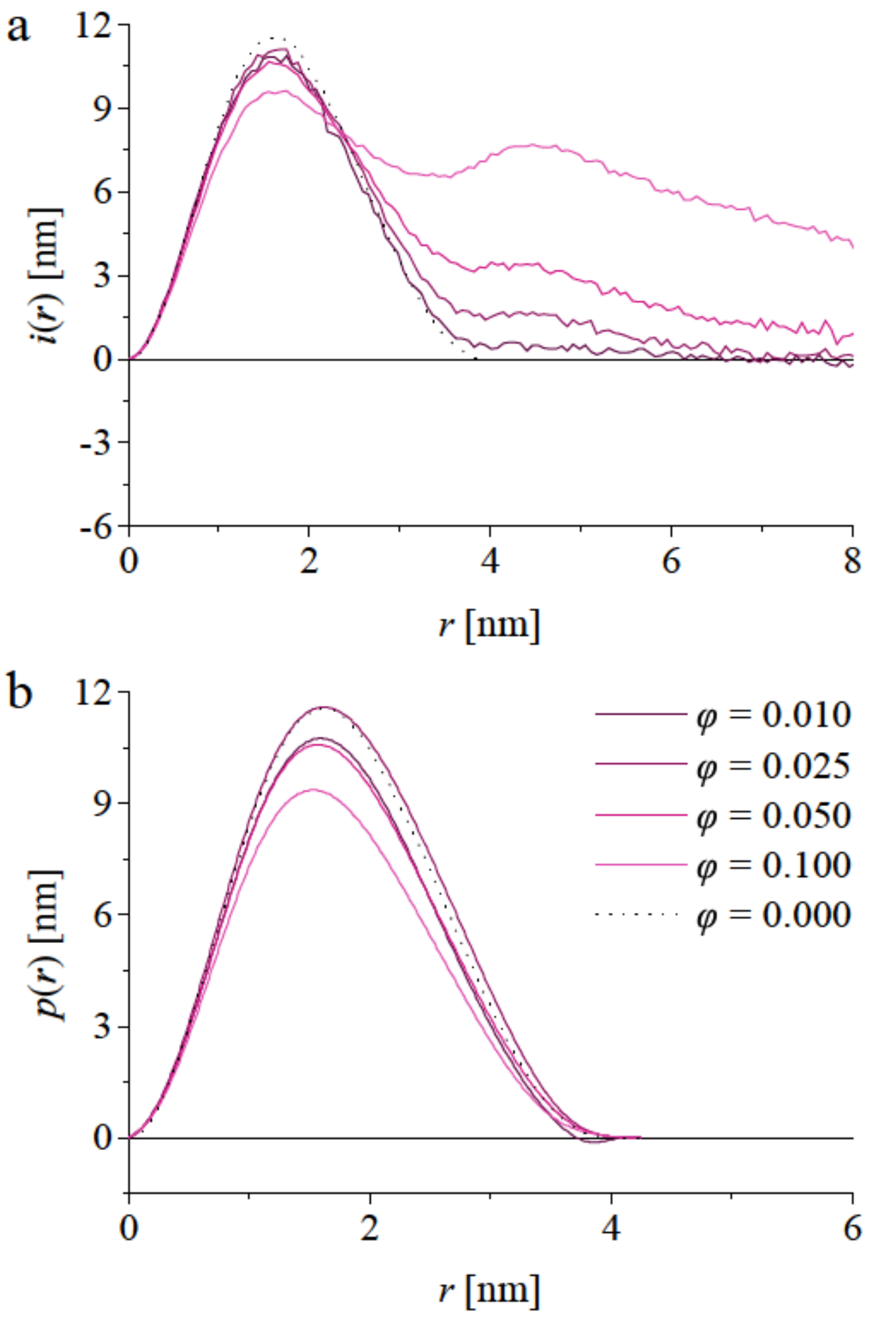

Figure SI–7 : Pair distance distribution functions of oblate ellipsoids with $\beta = -1.5\ kT$. a: Simulated functions, b: results from GIFT evaluation.

ure SI-8b. The shape of the pair distance distribution functions show little differences to the one expected for dilute ellipsoids. Therefore, the GIFT evaluations gives

Table SI–2 : Parameters obtained for the GIFT evaluation of scattering curves of oblate ellipsoids having attractive interaction of strength $\beta = -1.5\ kT$. φ: Simulated volume fraction, φ': volume fraction obtained from GIFT, r: particle radius, w: well width, β: well-depth

φ	φ'	r	w	β/kT
[%]	[%]	[nm]	[nm]	[ ]
1.0	2.0	1.65	0.09	-1.51
2.5	2.2	1.60	1.93	-0.73
5.0	0.8	1.24	1.13	-0.68
10.0	1.3	1.19	1.17	-0.07

reasonable results.

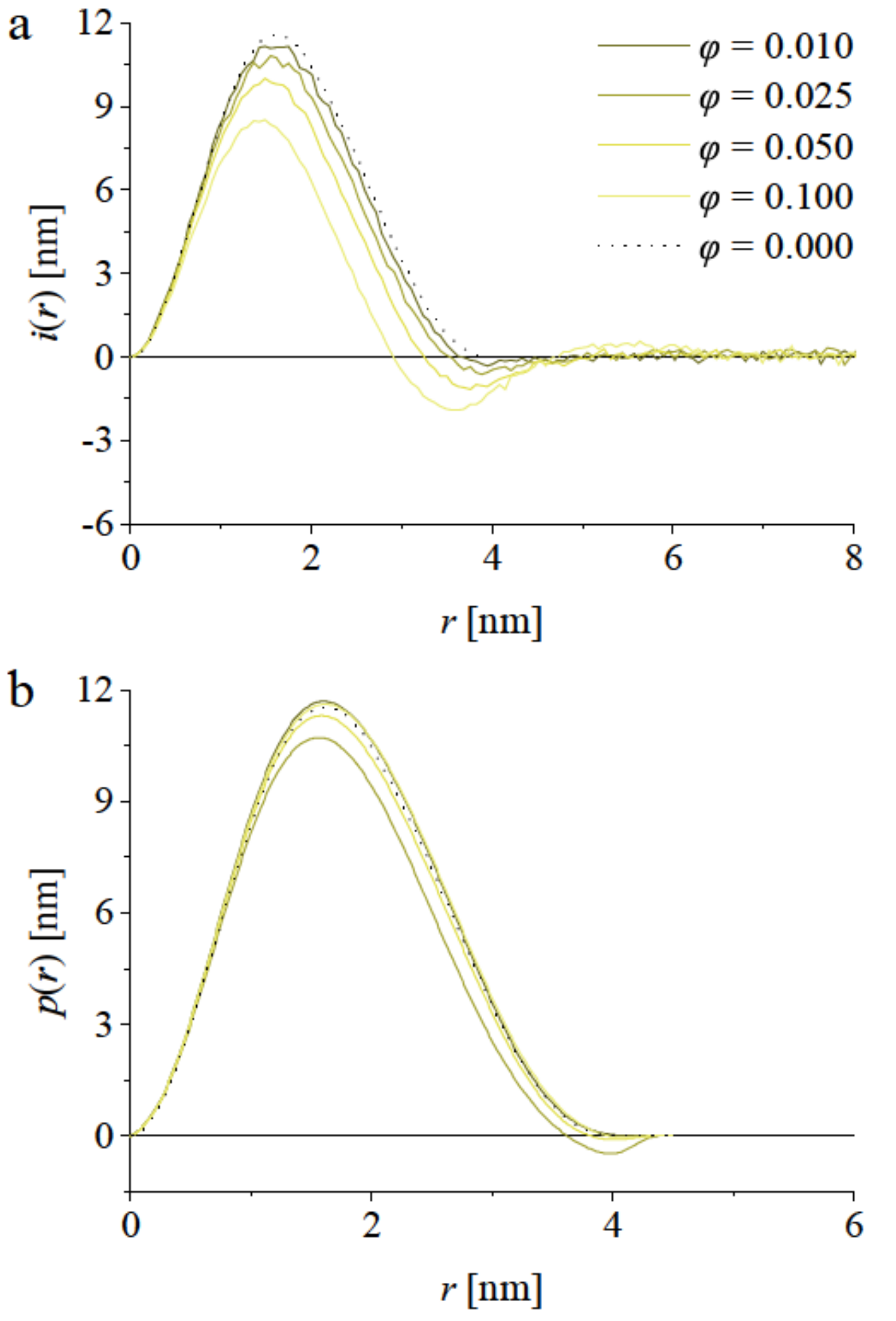

Figure SI–8 : Pair distance distribution functions of oblate ellipsoids with $\beta = -0.5\ kT$. a: Simulated functions, b: results from GIFT evaluation.

Table SI-3 gives the structure factor parameters obtained from GIFT. The volume fraction is generally underestimated. The interaction radius is between the lengths of the short and the long semi-axes of the ellipsoid and except for the lowest concentration rather consistent. The

Table SI–3 : Parameters obtained for the GIFT evaluation of scattering curves of oblate ellipsoids having attractive interaction of strength $\beta = -0.5\ kT$. φ: Simulated volume fraction, φ': volume fraction obtained from GIFT, r: particle radius, p: polydispersity

φ [%]	φ' [%]	r [nm]	p [ ]
1.0	1.0	1.22	0.000
2.5	2.4	1.49	0.098
5.0	3.6	1.43	0.000
10.0	8.3	1.42	0.001

difference at the lowest concentration is likely due to the fact that the weak interaction effects make the determination of any parameter based on them difficult.

5.3 *Oblate Ellipsoids with $\beta = +0.5\ kT$*

The $p(r)$ function of oblate particles at $\beta = +0.5\ kT$ are shown in figure SI-9a. They show minima close to the particle diameter and a small maximum at large distances. Consequently, the GIFT analysis was based on the averaged PY hard-sphere structure factor model. The resulting $p(r)$ functions are depicted in figure SI-9b. The shape of the resulting pair distance distribution functions is similar to the one expected for dilute ellipsoids.

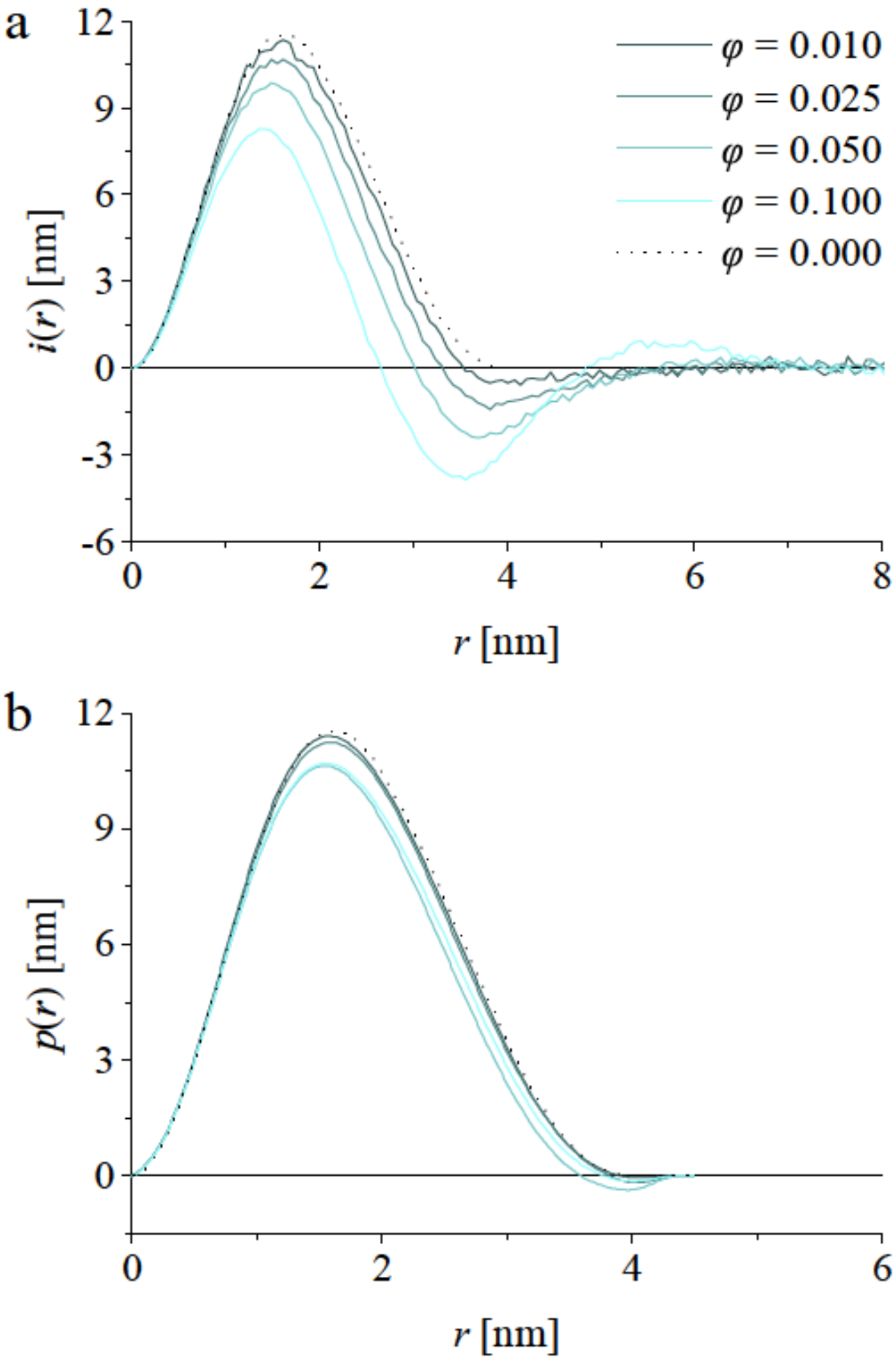

Figure SI–9 : Pair distance distribution functions of oblate ellipsoids with $\beta = +0.5\ kT$. a: Simulated functions, b: results from GIFT evaluation.

Table SI–4 : Parameters obtained for the GIFT evaluation of scattering curves of oblate ellipsoids having attractive interaction of strength $\beta = +0.5\ kT$. φ: Simulated volume fraction, φ': volume fraction obtained from GIFT, r: particle radius, p: polydispersity

φ [%]	φ' [%]	r [nm]	p [ ]
1.0	0.9	1.90	0.000
2.5	2.7	1.82	0.000
5.0	4.7	1.94	0.000
10.0	11.4	1.79	0.127

Table SI-4 gives the structure factor parameters obtained from GIFT. The values obtained for the volume fraction are approximately similar to the true volume fraction of the ellipsoids. The effective hard sphere radius is slightly smaller than the length of the long axes.

5.4 *Oblate Ellipsoids with $\beta = +1.5\ kT$*

The $p(r)$ function of oblate particles at $\beta = +1.5\ kT$ are shown in figure SI-10a. The minima and maxima at large distances indicate repulsive interactions, which typically can be described in terms of an effective hard sphere potential. Consequently, the GIFT analysis was based on the averaged PY hard-sphere structure factor model. The resulting $p(r)$ functions are depicted in figure SI-9b. The shape of the resulting pair distance distribution functions is similar to the one expected for dilute ellipsoids.

Table SI–5 : Parameters obtained for the GIFT evaluation of scattering curves of oblate ellipsoids having attractive interaction of strength $\beta = +1.5\ kT$. φ: Simulated volume fraction, φ': volume fraction obtained from GIFT, r: particle radius, p: polydispersity

φ [%]	φ' [%]	r [nm]	p [ ]
1.0	1.5	1.91	0.361
2.5	3.3	1.92	0.162
5.0	7.8	1.85	0.192
10.0	13.7	1.87	0.128

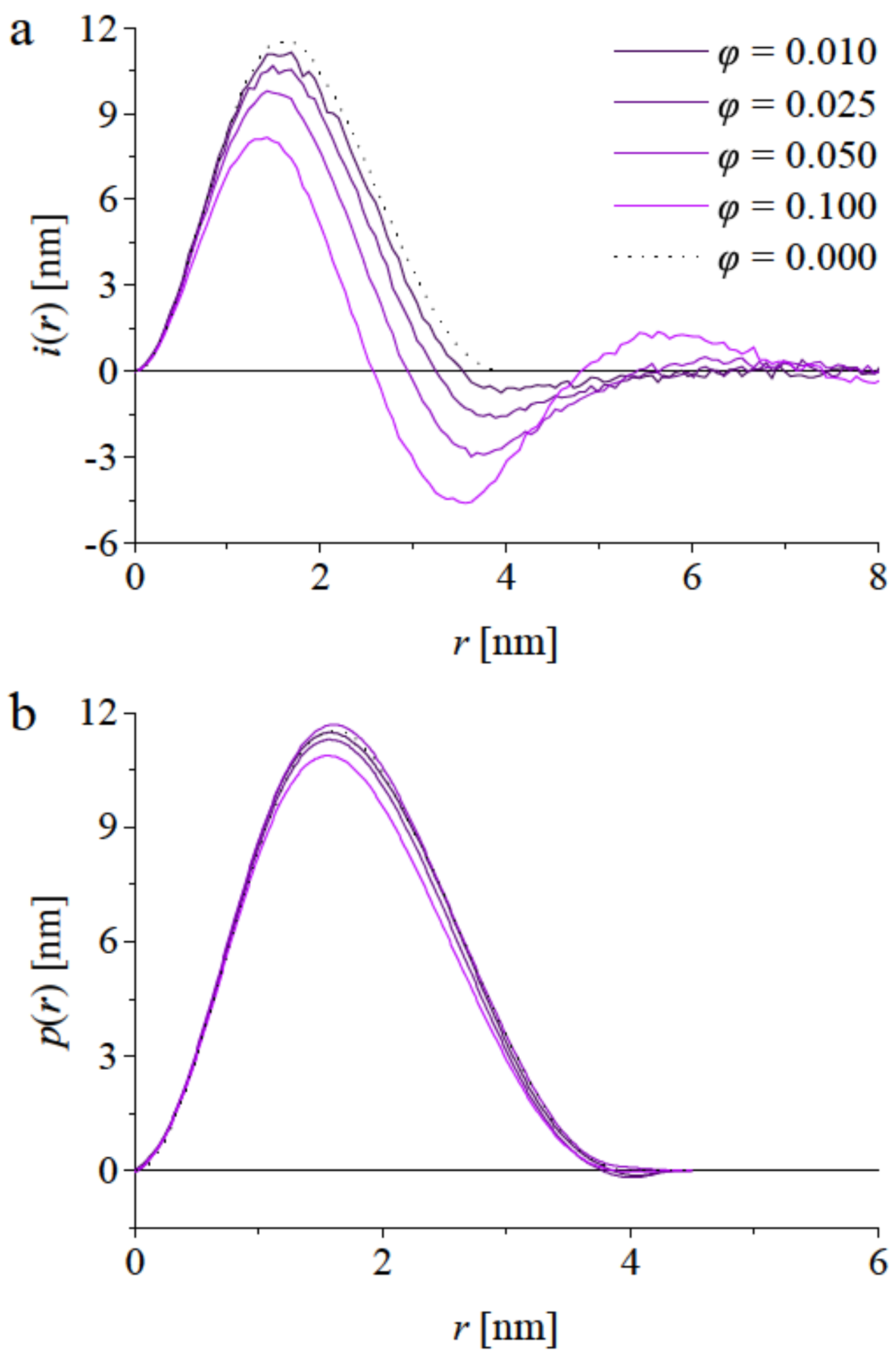

Figure SI–10 : Pair distance distribution functions of oblate ellipsoids with $\beta = +1.5\ kT$. a: Simulated functions, b: results from GIFT evaluation.

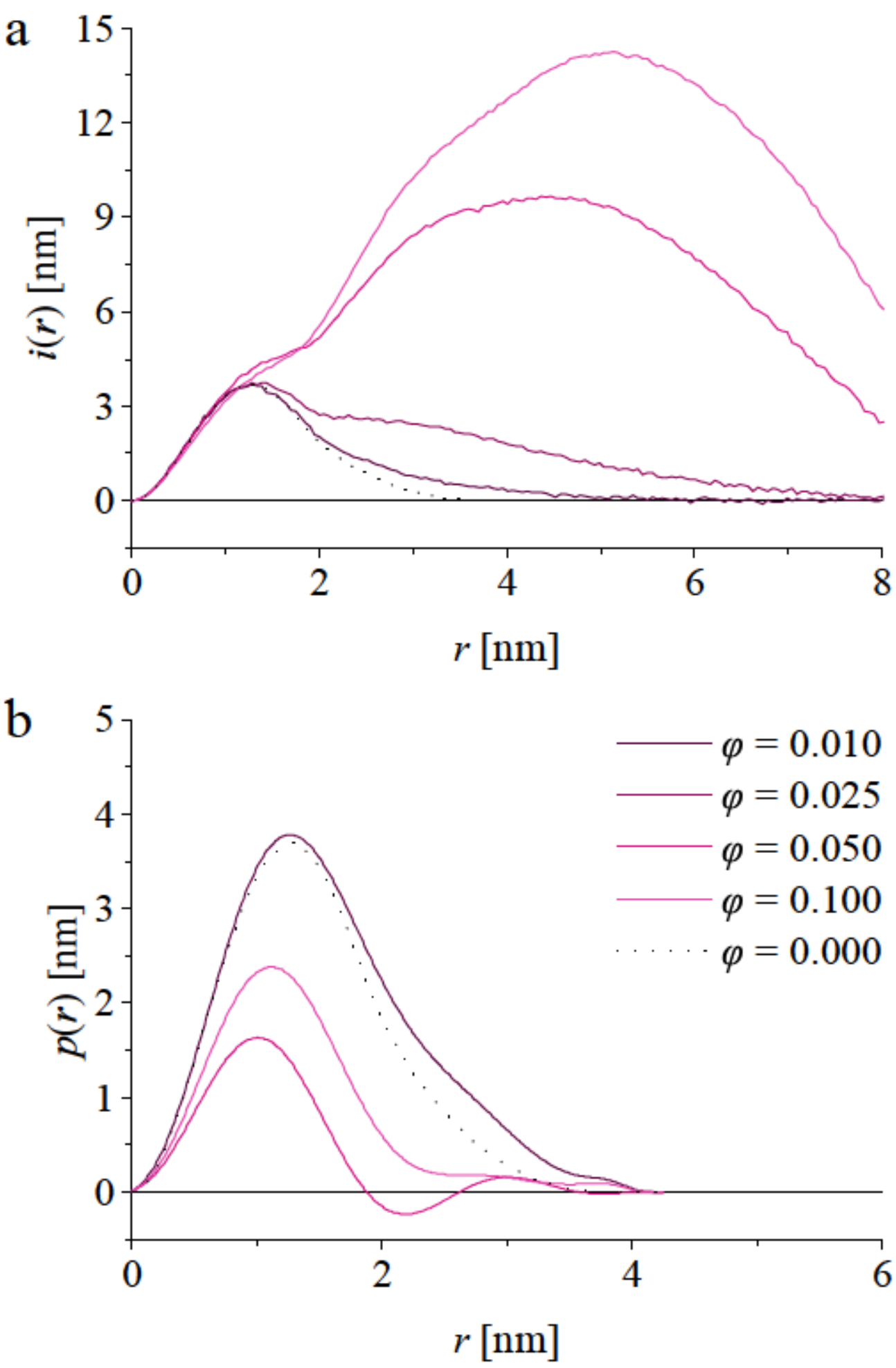

Figure SI–11 : Pair distance distribution functions of prolate ellipsoids with $\beta = -1.5\ kT$. a: Simulated functions, b: results from GIFT evaluation.

Table SI-5 gives the structure factor parameters obtained from GIFT. The volume fractions are obtained too high, which is consistent with the exclusion zone caused by the repulsive interactions. The effective radii are slightly smaller than the lengths of the long axes.

Table SI–6 : Parameters obtained for the GIFT evaluation of scattering curves of prolate ellipsoids having attractive interaction of strength $\beta = -1.5\ kT$. φ: Simulated volume fraction, φ': volume fraction obtained from GIFT, r: particle radius, w: well width, β: well-depth, r_0: characteristic size, d_f: fractal dimension, ξ: cut-off length

φ	φ'	r	w	β/kT
[%]	[%]	[nm]	[nm]	[ ]
1.0	1.5	1.37	0.44	-0.76
φ	r_0	d_f	ξ	-
[%]	[nm]	[ ]	[nm]	-
2.5	1.38	3.00	1.12	-
5.0	1.08	3.00	3.85	-
10.0	-	-	-	-

5.5 Prolate Ellipsoids with $\beta = -1.5\ kT$

The $p(r)$ function of prolate particles at $\beta = -1.5\ kT$ are shown in figure SI-11a. While the volume fraction of $\varphi = 0.01$ results in a function, which is similar to the one of single ellipsoids, all other $p(r)$-functions show the formation of clusters. These made GIFT evaluations very challenging.

The $\varphi = 0.010$ system could be evaluated using the SMSA closure relation in combination with a square well interaction potential. Using this approach for samples at higher volume fraction did not lead to any meaningful result. One might therefore try to apply the structure factor model for fractal aggregates [2].

The PDDF obtained by GIFT for the sample at volume fraction $\varphi = 2.5\%$ shows at least indications of an elongated particles (Fig. SI-11). However, the actual elliptical shape cannot be seen in this curve. The length of the long axis can be estimated, the short axes are slightly underestimated. Increasing the volume fraction to 5% results in a

Table SI–7 : Parameters obtained for the GIFT evaluation of scattering curves of prolate ellipsoids having attractive interaction of strength $\beta = -0.5\ kT$. φ: Simulated volume fraction, φ': volume fraction obtained from GIFT, r: particle radius, w: well width, β: well-depth

φ [%]	φ' [%]	r [nm]	w [nm]	β/kT [ ]
1.0	0.1	1.81	0.52	-0.94
2.5	0.1	1.94	1.93	-0.32
5.0	0.2	1.31	0.93	-0.46
10.0	0.6	1.33	0.92	-0.40

PDDF from GIFT, which cannot be interpreted correctly, while 10% did not lead to any stable result. These findings are also reflected by the parameters obtained for the structure factor models (Table SI-6).

5.6 *Prolate Ellipsoids with* $\beta = -0.5\ kT$

Approximations of the scattering curves using the GIFT in combination with the SMSA structure factor model for square-well particles were also done. The resulting $p(r)$ basically did not show any significant difference to the simulated functions (Main text, figure 4), which indicates that interaction effects were not taken into account correctly.

Table SI-7 gives the corresponding structure factor parameters obtained by GIFT. The evaluation results in some kind of attractive interaction, but the parameters for particle radius, well width and well depth are not stable. Only the volume fraction is consistently low. Actually, it is too low by a factor of 10 to 25 thereby basically eliminating most of the structure factor effects from the calculation.

5.7 *Prolate Ellipsoids with* $\beta = +0.5\ kT$

The PDDFs of prolate ellipsoids having repulsive interactions at $\beta = +0.5\ kT$ are shown in figure SI-12a. They show minima and maxima, which are slightly more pronounced than in the case of ellipsoids interacting by excluded volume effects only.

Figure SI-12b depicts the PDDFs obtained for these sampls by GIFT using the averaged hard sphere structure factor model. The corresponding structure factor parameters are given in Table SI-8. The PDDFs are those expected for such ellipsoids. The structure factor parameters are dominated mostly by the large volume polydispersity, whereby the $S(q)$ becomes largely featureless except for broad a minimum at $q = 0$. This minimum is quite pronounced, since the repulsive interaction leads to an increased effective volume fraction.

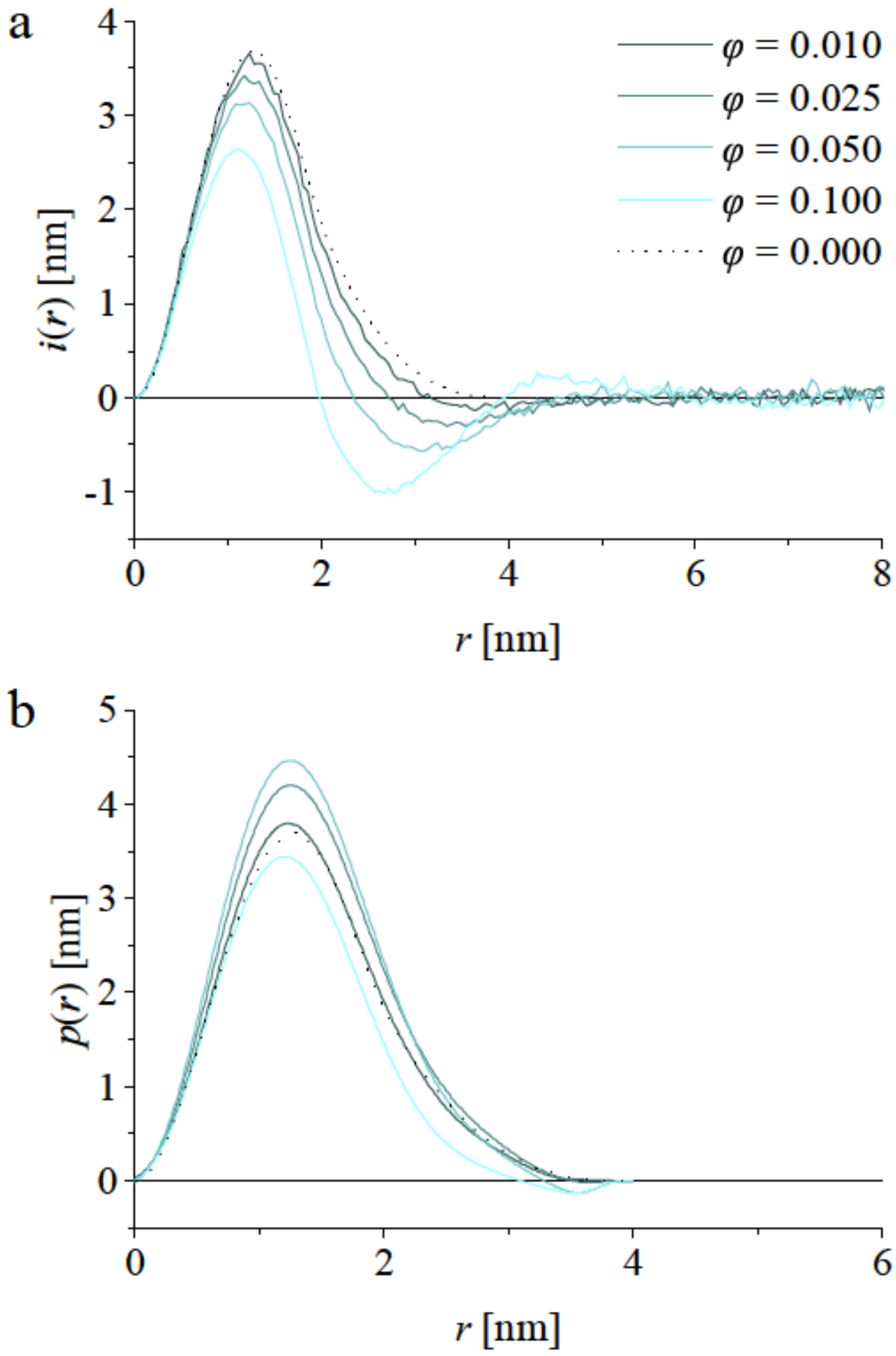

Figure SI–12 : Pair distance distribution functions of prolate ellipsoids with $\beta = +0.5\ kT$. a: Simulated functions, b: results from GIFT evaluation.

Table SI–8 : Parameters obtained for the GIFT evaluation of scattering curves of prolate ellipsoids having attractive interaction of strength $\beta = +0.5\ kT$. φ: Simulated volume fraction, φ': volume fraction obtained from GIFT, r: particle radius, p: polydispersity

φ [%]	φ' [%]	r [nm]	p [ ]
1.0	1.5	1.08	0.962
2.5	5.2	1.00	0.744
5.0	9.0	1.01	0.735
10.0	11.3	1.39	0.211

5.8 *Prolate Ellipsoids with* $\beta = +1.5\ kT$

Figure SI-13a shows the PDDFs of prolate ellipsoids at $\beta = +1.5\ kT$. The minima and maxima follow the same trend as in the case of $\beta = +0.5\ kT$.

GIFT analysis results in PDDFs that are also in line with the ones expected for such ellipsoids (Figure SI-

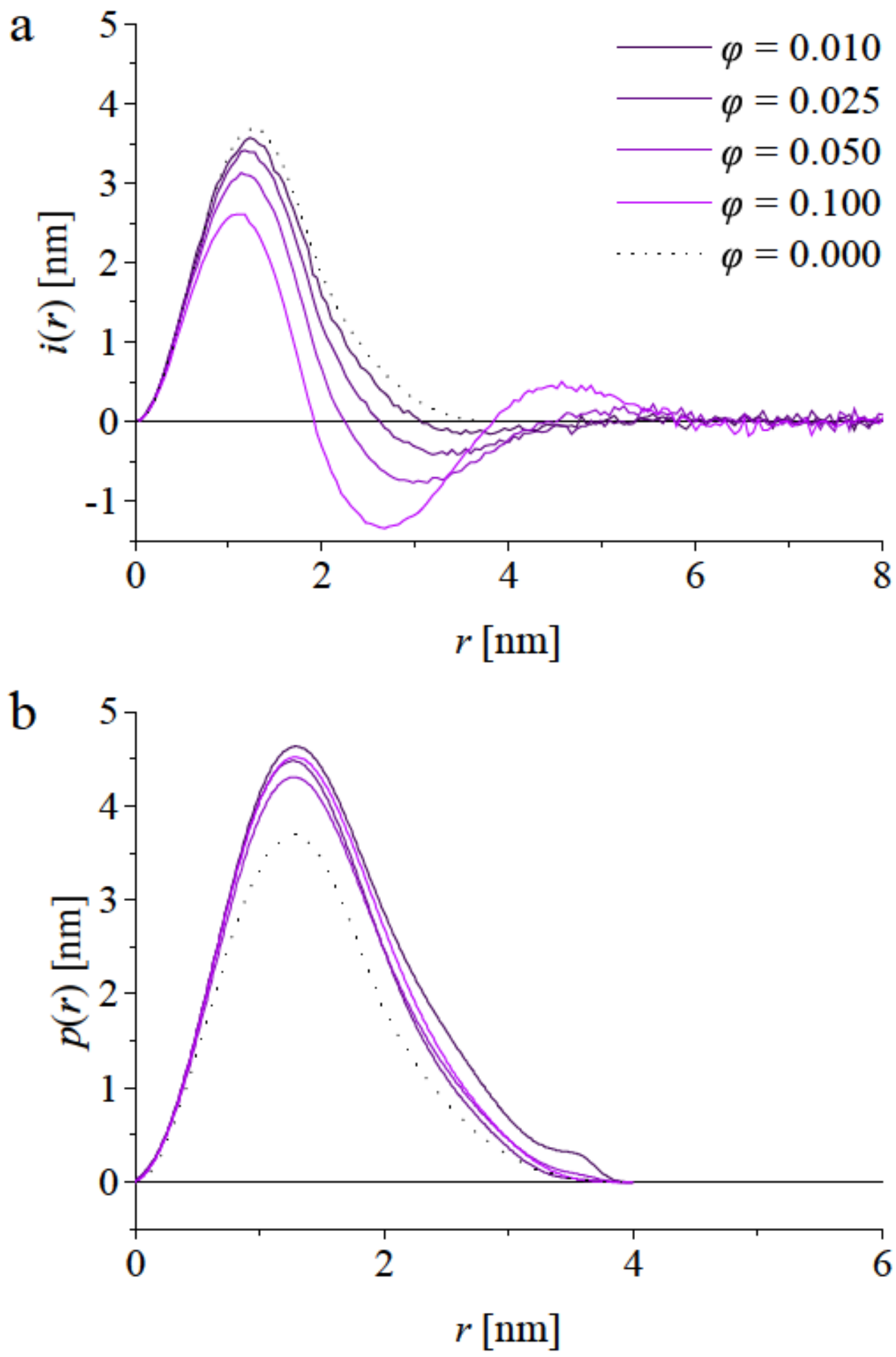

Figure SI–13 : Pair distance distribution functions of prolate ellipsoids with $\beta = +1.5\ kT$. a: Simulated functions, b: results from GIFT evaluation.

Table SI–9 : Parameters obtained for the GIFT evaluation of scattering curves of prolate ellipsoids having attractive interaction of strength $\beta = +1.5\ kT$. φ: Simulated volume fraction, φ': volume fraction obtained from GIFT, r: particle radius, p: polydispersity

φ [%]	φ' [%]	r [nm]	p [ ]
1.0	5.8	1.00	0.532
2.5	6.9	1.00	0.822
5.0	10.8	1.37	0.339
10.0	18.7	1.37	0.275

13b). The corresponding structure factor parameters (Table SI-9) proves that the results are similar to the ones obtained for $\beta = +0.5\ kT$ with the expected difference of a higher effective volume fraction.

Table SI–10 : Parameters derived through the approximation of an ellipsoid of revolution to scattering curves of prolate ellipsoids having attractive interaction of strength $\beta = -0.5\ kT$. φ: Simulated volume fraction, a: single half-axis, b: double half-axis, $\Delta\rho/\Delta\rho_0$: contrast

φ [%]	a [nm]	b [nm]	$\Delta\rho/\Delta\rho_0$ [ ]
1.0	1.97 ± 0.02	1.00 ± 0.01	0.99 ± 0.01
2.5	1.88 ± 0.03	0.99 ± 0.01	1.02 ± 0.00
5.0	1.76 ± 0.03	0.98 ± 0.01	1.05 ± 0.01
10.0	1.47 ± 0.01	0.97 ± 0.00	1.12 ± 0.00

6 Fits to Scattering Data of Prolate Ellipsoids at $\beta = -0.5\ kT$

The scattering curves of prolate ellipsoids simulated with an attractive interaction of $\beta = -0.5\ kT$ did not exhibit clear indications of interaction effects. Consequently, an attempt was made to describe them using a model consisting solely of the pure form factor of an ellipsoid of revolution. The resulting fit parameters are presented in Table SI-10. It is worth to notice that the model does not take interaction effects into account.

7 Simulated Scattering Curves

The simulated scattering curves and the corresponding approximations obtained by GIFT are shown in figures 14 to 20.

Note that the simulated scattering curves of a single particle shape show some differences between each other, which are unlikely to be due to interaction effects. They also deviate there most from the theoretical scattering curves (Fig. 1 of the main text). These effects are explained best by some effective surface roughness introduced by the Monte Carlo integration process leading to the $i(r)$ functions.

8 SAXS Experiments on Lysozyme

8.1 Approximation of Lysozyme Structure by Ellipsoid

The scattering curve and the pair distance distribution function of lysozyme has been calculated using a bead model [3] based on a crystal structure [4]. The scattering curve was then approximated by the scattering curve of an ellipsoid of revolution resulting in resulting in a single long axis of 2.62 nm and the short double axes of 1.54 nm. These data were used to calculate [5] the scattering curve and the pair distance distribution function of such an ellipsoid.

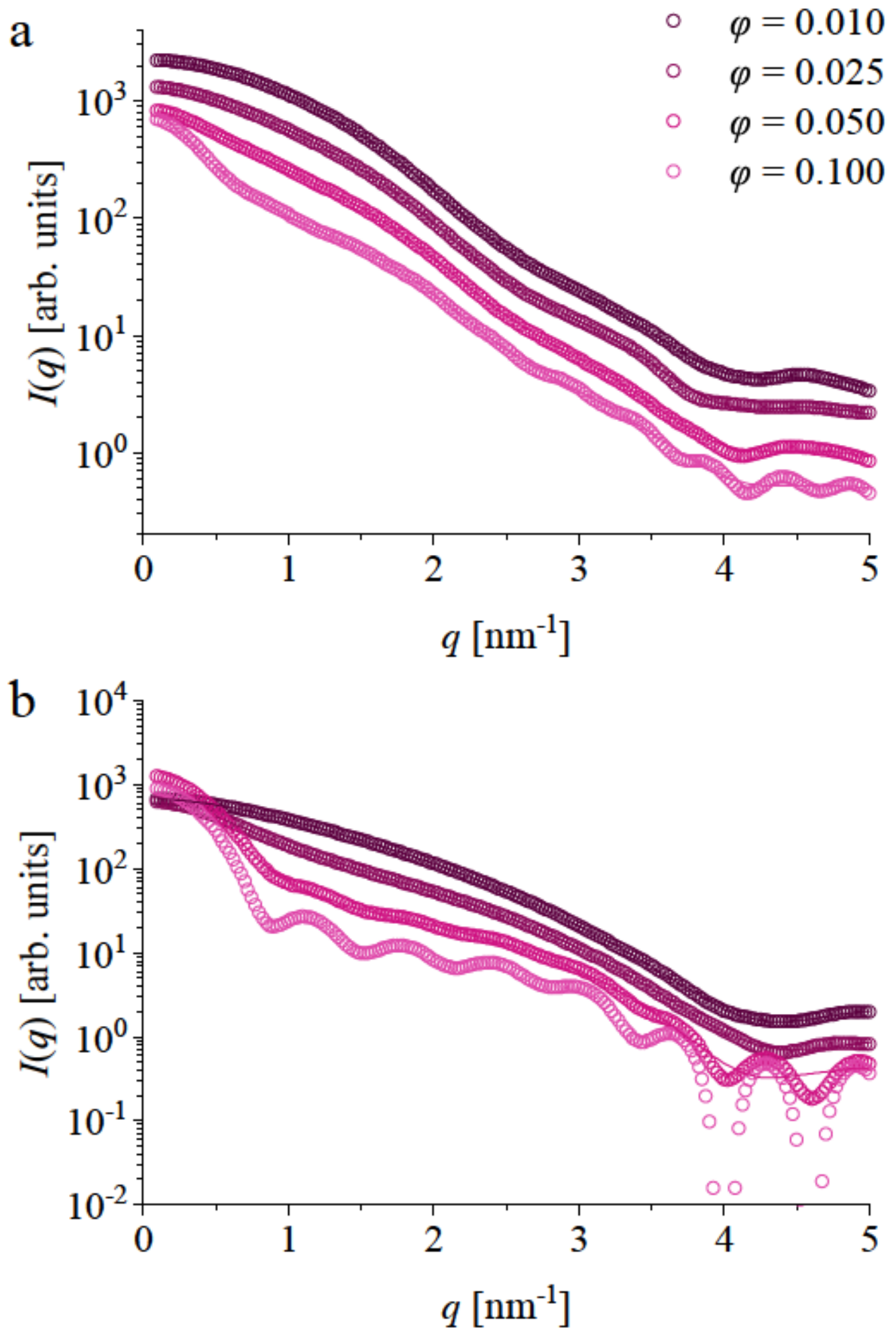

Figure SI–14 : Simulated scattering curves (symbols) and approximations obtained by GIFT (lines) at $\beta = -1.5\,kT$. a: Oblate ellipsoids, b: Prolate ellipsoids.

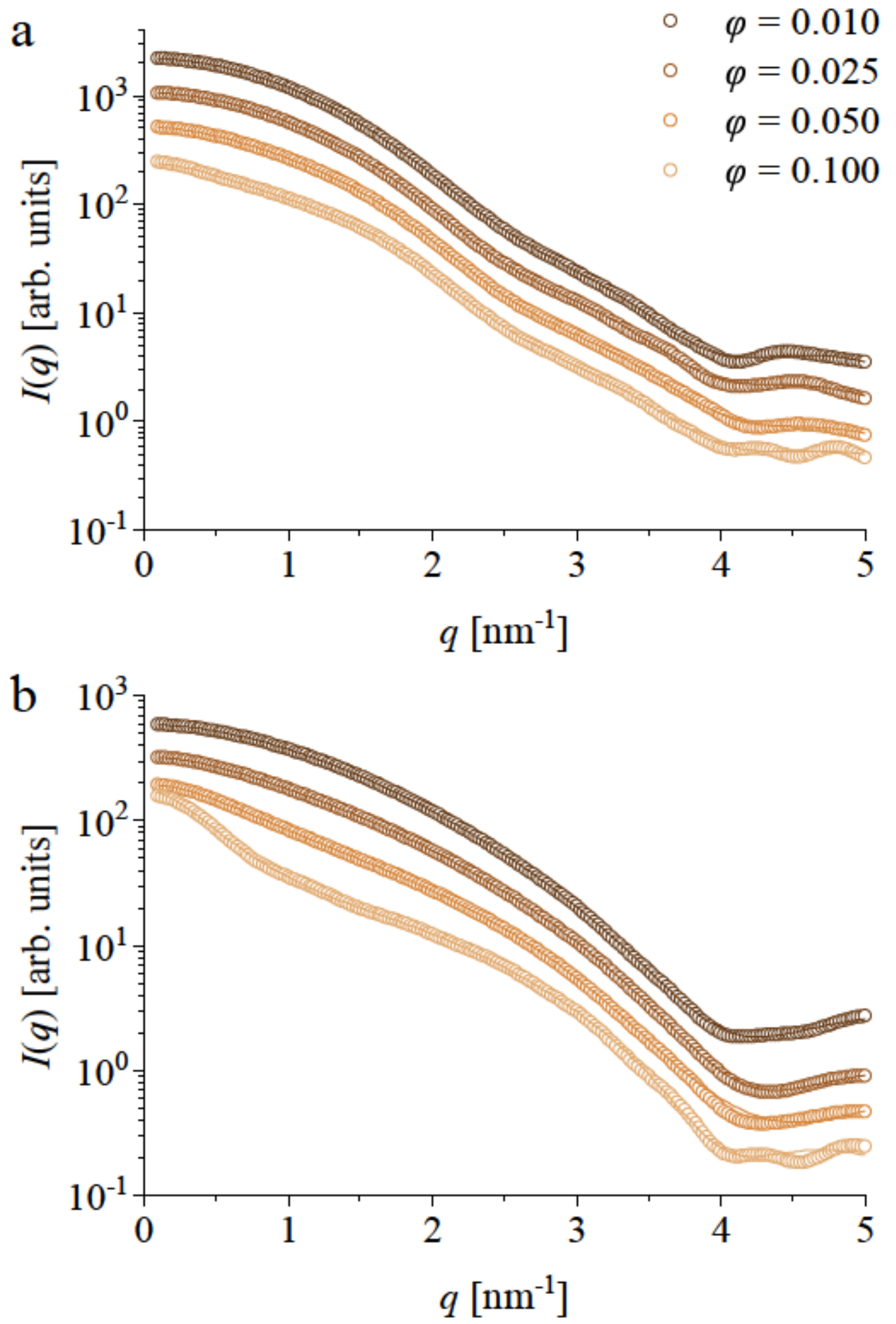

Figure SI–15 : Simulated scattering curves (symbols) and approximations obtained by GIFT (lines) at $\beta = -1.0\,kT$. a: Oblate ellipsoids, b: Prolate ellipsoids.

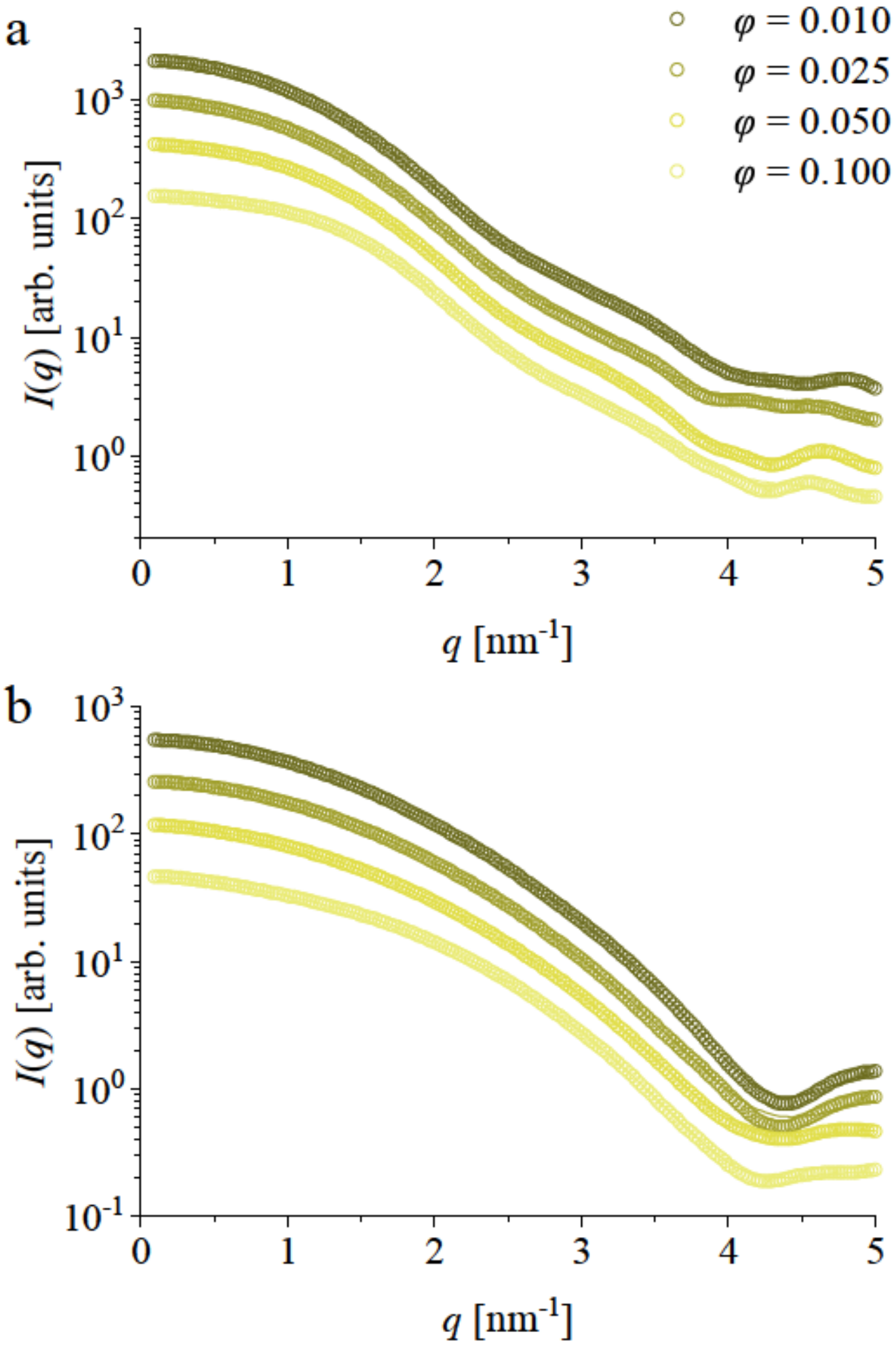

Figure SI–16 : Simulated scattering curves (symbols) and approximations obtained by GIFT (lines) at $\beta = -0.5\,kT$. a: Oblate ellipsoids, b: Prolate ellipsoids.

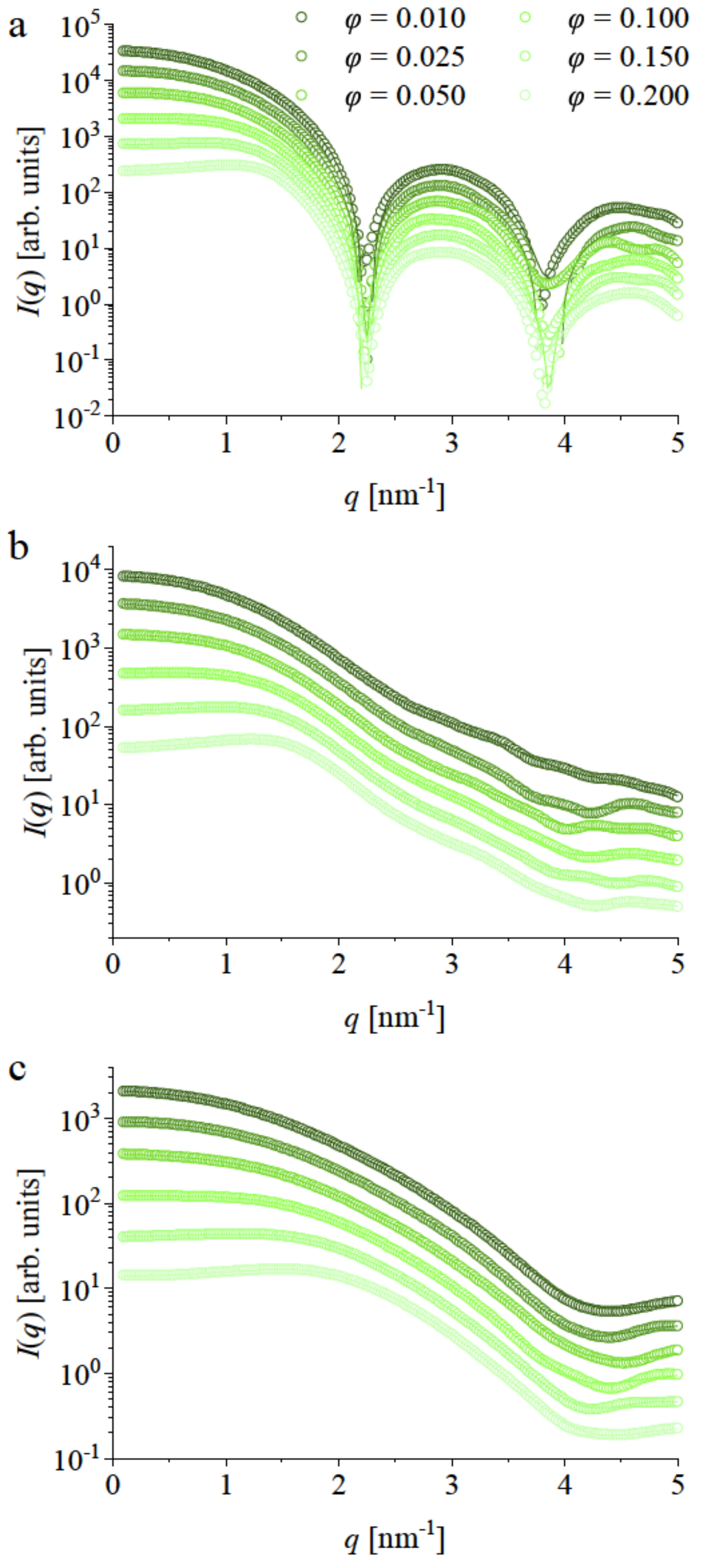

Figure SI–17 : Simulated scattering curves (symbols) and approximations obtained by GIFT (lines) at $\beta = 0$. a: Spheres, b: Oblate ellipsoids, c: Prolate ellipsoids.

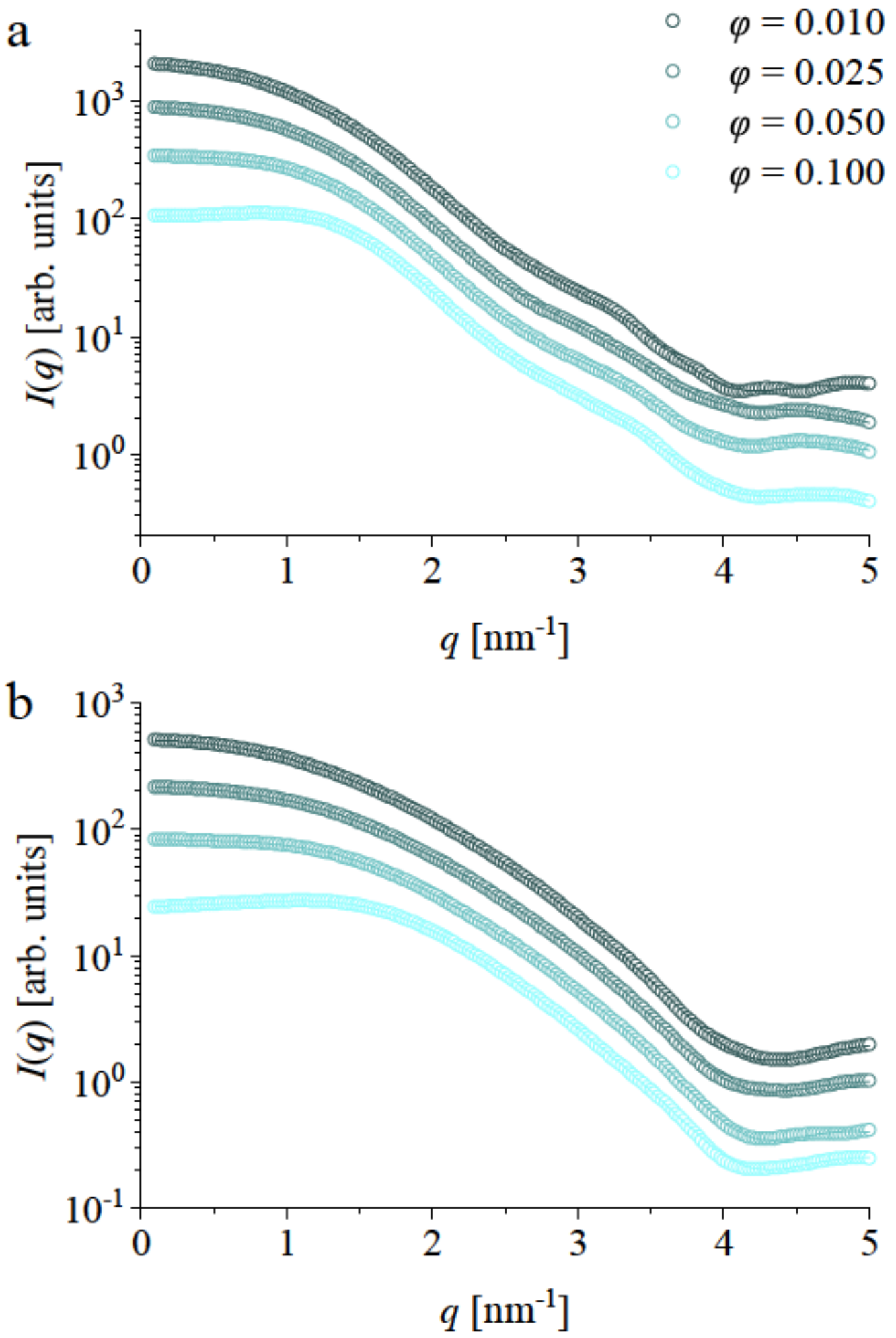

Figure SI–18 : Simulated scattering curves (symbols) and approximations obtained by GIFT (lines) at $\beta = +0.5\,kT$. a: Oblate ellipsoids, b: Prolate ellipsoids.

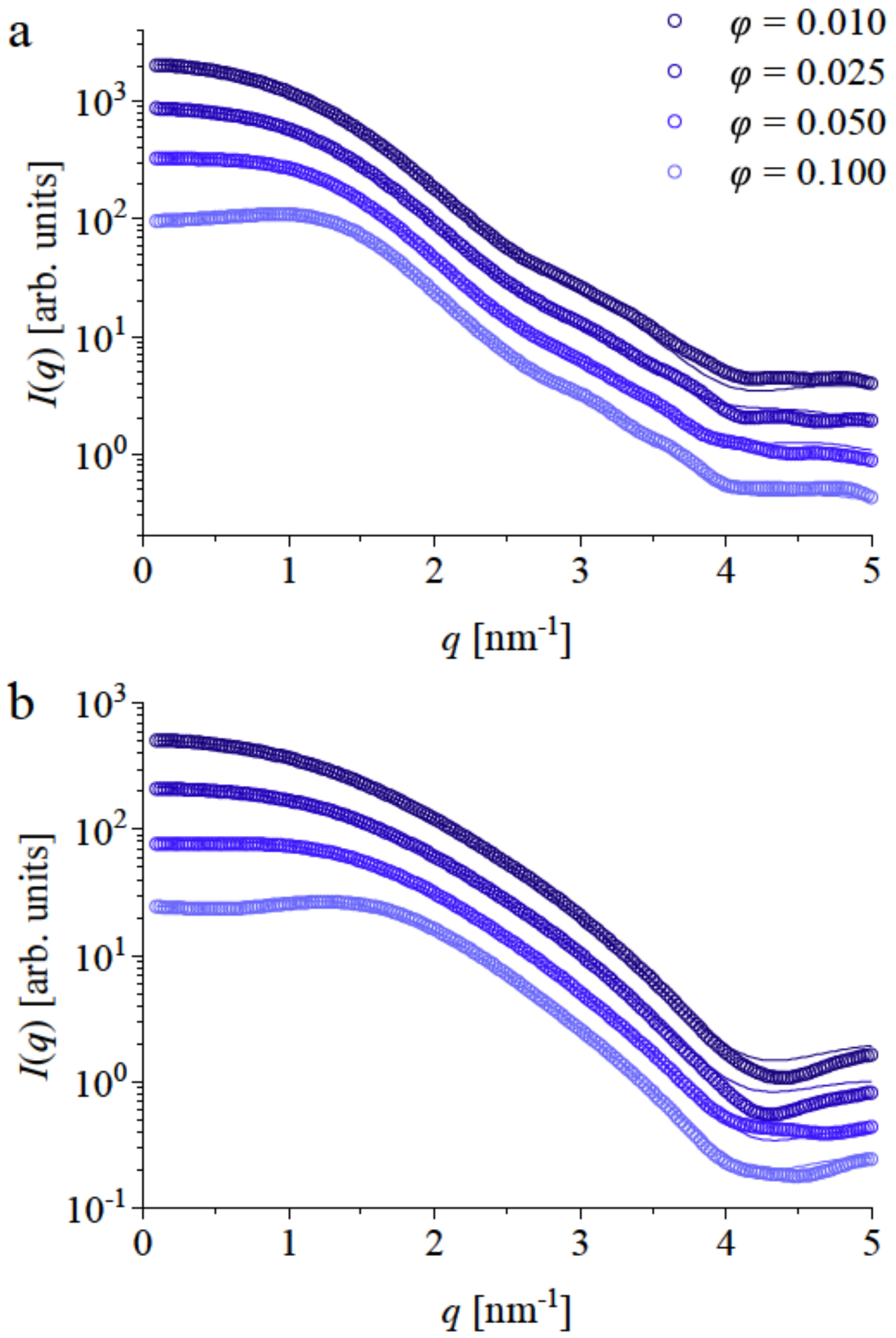

Figure SI–19 : Simulated scattering curves (symbols) and approximations obtained by GIFT (lines) at $\beta = +1.0\,kT$. a: Oblate ellipsoids, b: Prolate ellipsoids.

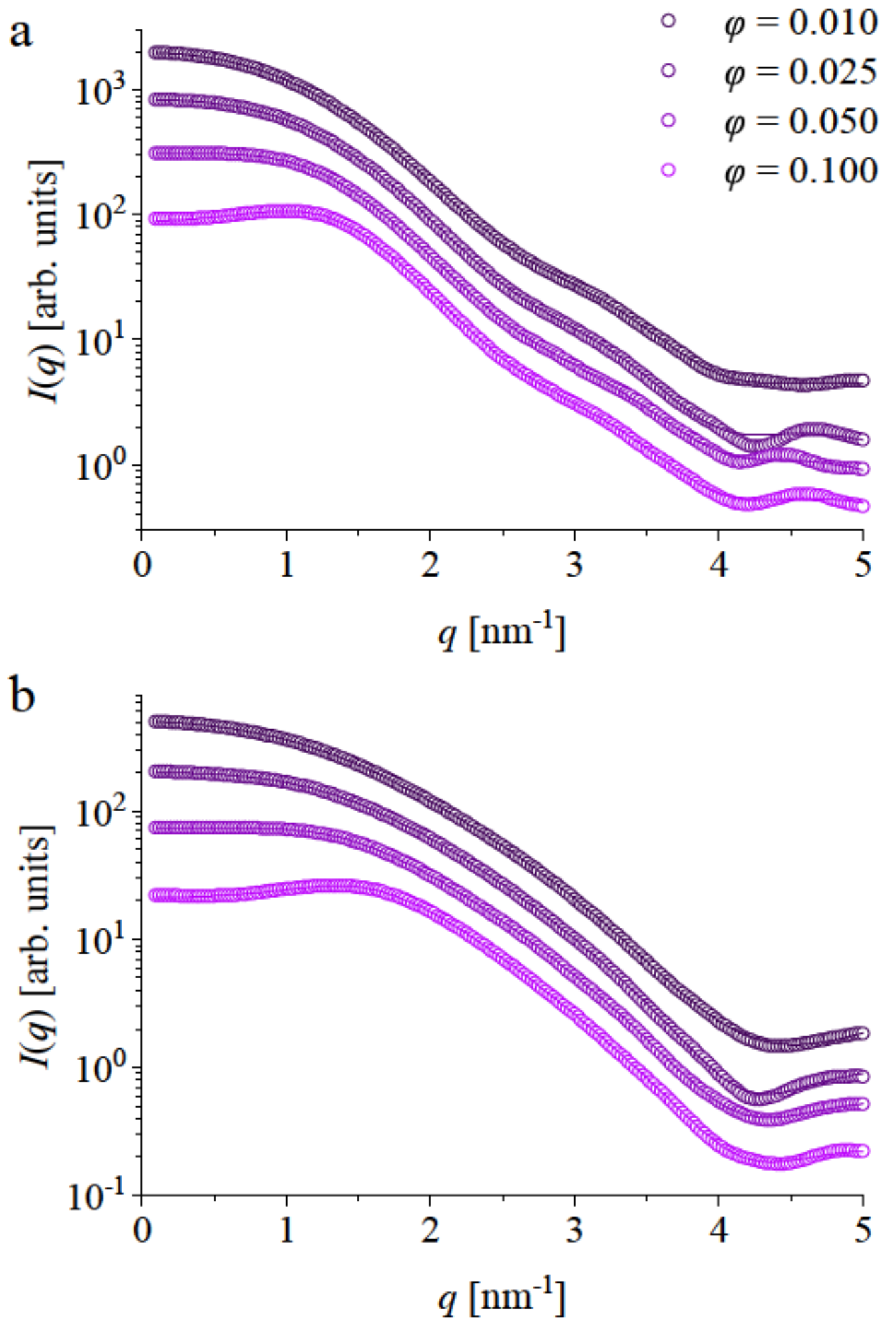

Figure SI–20 : Simulated scattering curves (symbols) and approximations obtained by GIFT (lines) at $\beta = +1.5\,kT$. a: Oblate ellipsoids, b: Prolate ellipsoids.

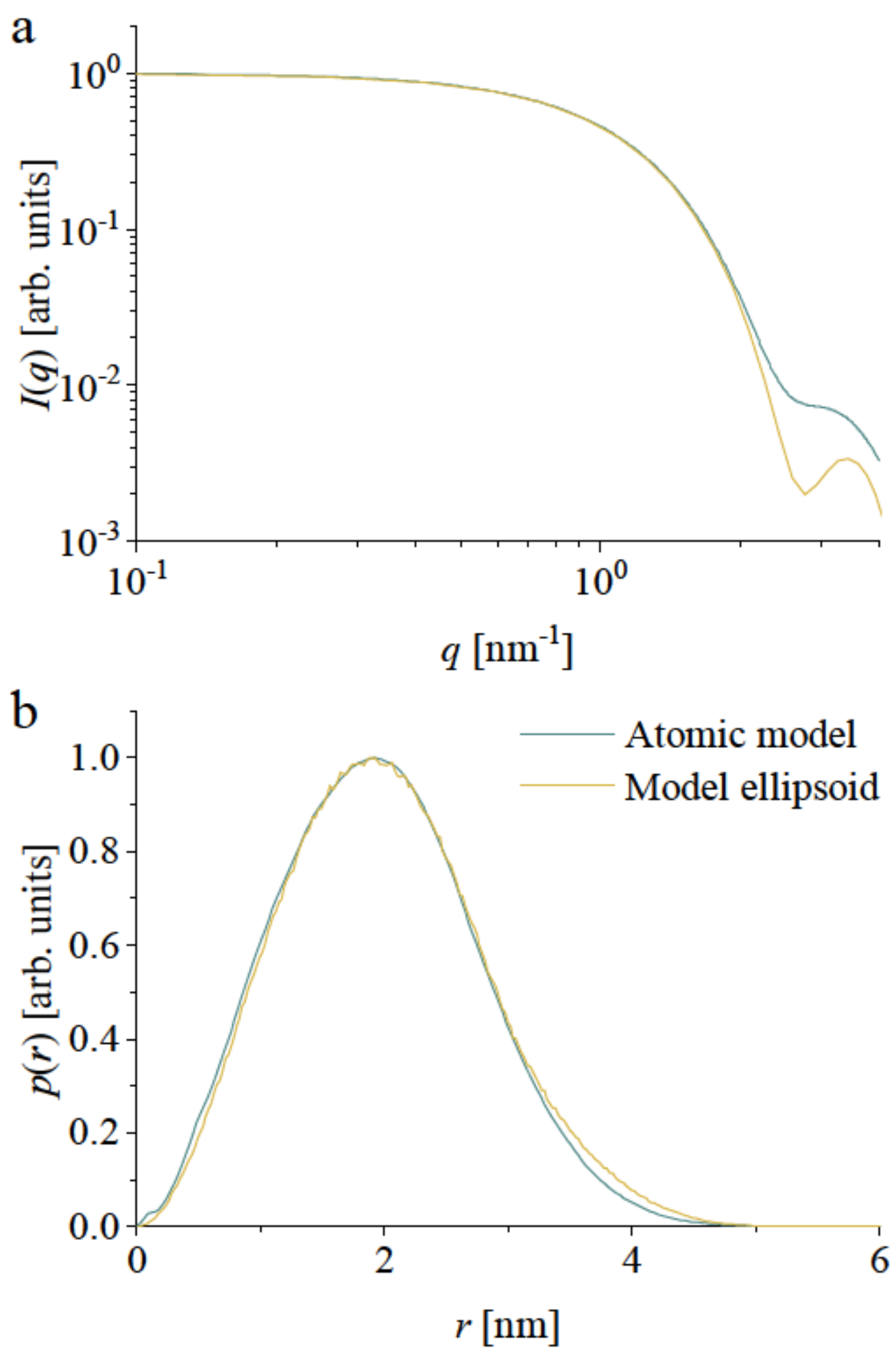

Figure SI–21 : a: Scattering curve of lysozyme based on crystallographic data and best fit of ellipsoid of revolution. b: Corresponding pair distance distribution functions.

Table SI–11 : Parameters derived through the approximation of an ellipsoid of revolution to scattering curves of lysozyme in 0.19 mol/l NaCl solutions. *c*: concentration (w/w), *a*: single half-axis, *b*: double half-axis, $\Delta\rho$: contrast

c [%]	a [nm]	b [nm]	$\Delta\rho/\Delta\rho_0$ [arb. units]
1.0	2.52 ± 0.02	1.43 ± 0.01	0.267 ± 0.001
2.5	2.34 ± 0.02	1.47 ± 0.01	0.409 ± 0.001
5.0	2.29 ± 0.02	1.45 ± 0.01	0.843 ± 0.002
9.9	1.85 ± 0.01	1.58 ± 0.00	0.934 ± 0.003

Figure 21 shows these curves. Differences in the scattering curves at $q \approx 2.5$ nm^{-1} indicate mainly the higher symmetry of the ellipsoid, *i.e.* the local deviations of the protein from ellipsoidal shape. The similarity of the PDDF_p functions demonstrate that the prolate ellipsoid of revolution is a reasonable approximation for the overall structure of the protein.

8.2 PDDFs of Aqueous Lysozyme Solutions

The scattering curves and the corresponding $p(r)$ functions in Figure SI-22 have not been scaled. The vertical differences are primarily due to varying concentrations.

The scattering curves of lysozyme solutions in pure water (Figure SI-22a) exhibit clear interaction peaks, which shift to larger q-values with increasing concentration. This behaviour is consistent with the expectation for strong repulsive interactions. This is reflected by the strong oscillations of the $p(r)$ functions obtained by the IFT (Figure SI-22b).

Electrostatic screening through the addition of salt (0.10 mol/l) reduces the repulsive interaction. The scattering curves no longer display the pronounced peaks observed in the solutions in pure water (Figure SI-22c), although higher concentrations lead to the onset of the formation of such a peak. Consequently, the long-range order is less pronounced, which is reflected by the stronger damping of the oscillations in the $p(r)$ function at larger distances (Figure SI-22d).

A further increase in the salt concentration to 0.19 mol/l screens the electrostatic interactions even more. The scattering curves shift with increasing concentration (Figure SI-22e), but no interaction peak is observed. The real-space analogue obtained by IFT shows negligible traces of interaction effects (Figure SI-22f).

8.3 Results of GIFT Evaluation

The $p(r)$ functions obtained by the GIFT evaluation of the scattering curves of lysozyme are shown in figure 7 of the main text. The corresponding structure factor parameters obtained for the averaged hard sphere structure factor model are given in tables 12, 13, and 14.

The structure factor parameters indicate strong repulsive behaviour in pure water, which leads to volume fractions that are considerably higher than the weight fractions of the proteins. Adding 100 mmol/L of NaCl reduces electrostatic repulsion, but some are still present, which can be described by hard spheres at concentrations, which are on the same order of magnitude as the actual ones. This apparent volume fraction stays constant at higher concentrations. This might be linked to the problems of the GIFT technique, to reproduce the $p(r)$ of a prolate ellipsoid for these samples. The PDDF_p of these samples looks more similar to the one of spheres (Main

Table SI–12 : Structure factor parameters obtained by GIFT for lysozyme in pure water.

c [%]	φ' [%]	r [nm]	p [ ]
1.0	12.4	5.05	0.254
2.5	17.8	3.78	0.281
5.0	20.3	3.07	0.252
10.0	20.3	2.60	0.193
15.0	19.4	2.41	0.173
19.9	18.8	2.21	0.173

Table SI–13 : Structure factor parameters obtained by GIFT for lysozyme in 100 mmol/L NaCl solution.

c [%]	φ' [%]	r [nm]	p [ ]
1.0	0.1	4.94	0.004
2.5	1.2	2.22	0.029
5.0	5.0	2.06	0.193
10.0	7.4	1.98	0.300
15.0	7.5	2.06	0.231
19.9	7.8	1.87	0.300

Table SI–14 : Structure factor parameters obtained by GIFT for lysozyme in 200 mmol/L NaCl solution.

c [%]	φ' [%]	r [nm]	p [ ]
1.0	1.5	7.59	0.066
2.5	3.4	8.27	0.023
5.0	1.2	2.33	0.002
10.0	1.3	2.05	0.092

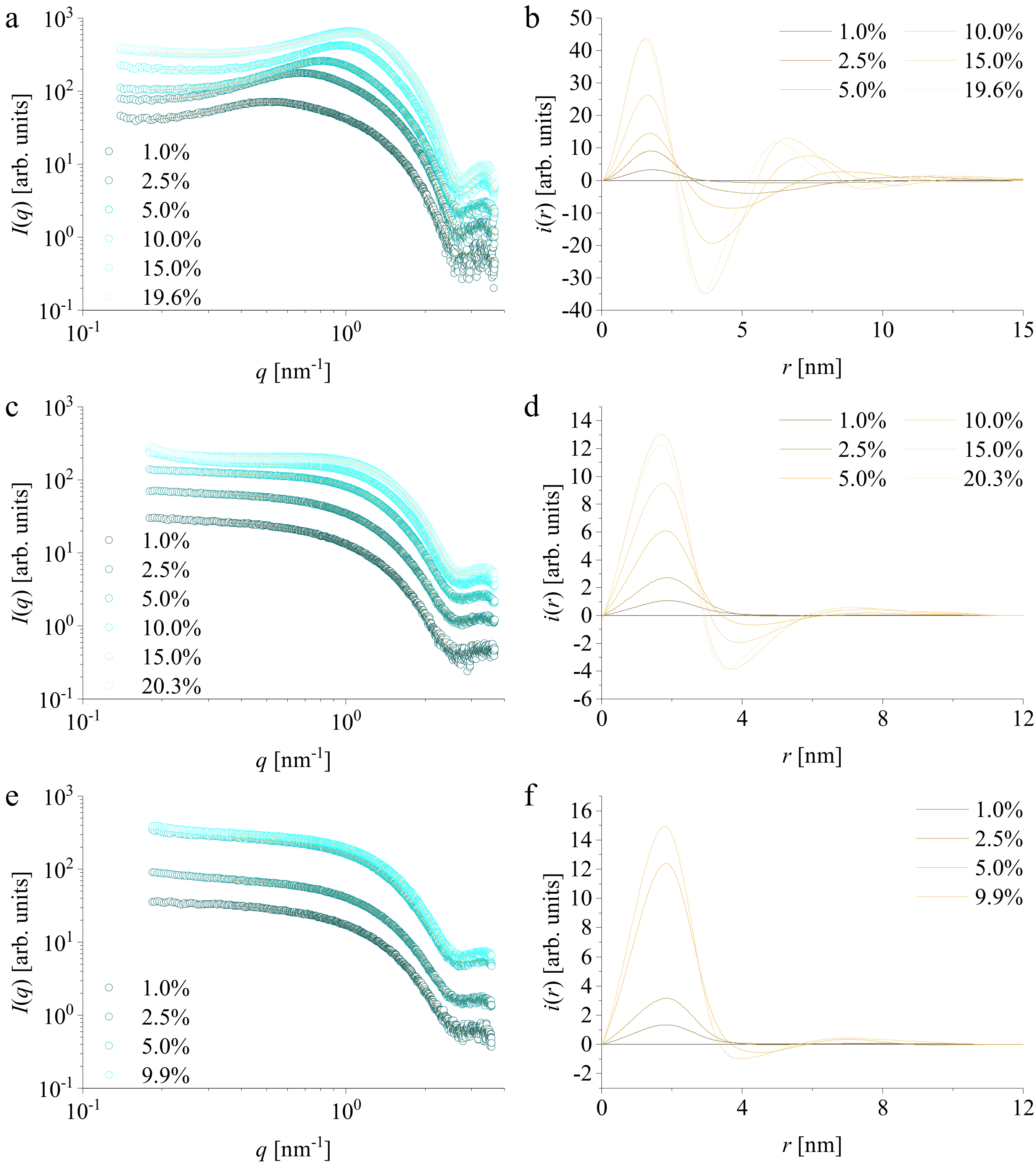

Figure SI–22 : a: Scattering curves of lysozyme solutions in deionized water, b: corresponding pair distance distribution functions. c, d: scattering curves and corresponding PDDFs of lysozyme in aqueous 0.10 mol/l NaCl solutions. e, f: scattering curves and corresponding PDDFs of lysozyme in aqueous 0.19 mol/l NaCl solutions. The yellowisch curves in a, c, and d are the approximations obtained by IFT.

Table SI–15 : Single axis (a) and double axes b obtained by approximation of ellipsoid to GIFT results.

	0 mM		100 mM		200 mM	
c	a	b	a	b	a	b
[%]	[nm]	[nm]	[nm]	[nm]	[nm]	[nm]
1.0	2.45	1.49	2.44	1.58	2.44	1.42
2.5	2.55	1.50	2.46	1.48	2.29	1.45
5.0	2.60	1.49	2.56	1.47	2.32	1.43
10.0	2.34	1.49	2.32	1.49	1.91	1.54
15.0	2.10	1.49	2.05	1.48	-	-
≈20	2.00	1.49	1.98	1.49	-	-

text, figure 7d). The structure factor parameters obtained for the curves measured at 200 mmol/L NaCl all show low apparent volume fractions. This is consistent with the apparent lack of interaction effects.

Table 15 gives the parameters, which are obtained by approximating the model of an ellipsoid of revolution to the GIFT results. While the short axes are found to be 1.5 ± 0.1 nm, the long axis has a tendency to be shorter values with increasing concentration. This is in line with the shape of the $p(r)$ functions, which would be interpreted to be closer to a sphere with increasing concentration.

References

[1] André Guinier and Gérard Fournet. *Small-Angle Scattering of X-Rays*. John Wiley & Sons, New York, 1955.

[2] J. Teixeira. Small-angle scattering by fractal systems. *J. Appl. Crystallogr.*, 21:781–785, 1988.

[3] Otto Glatter. Computation of distance distribution functions and scattering functions of models for small angle scattering experiments. *Acta Phys. Aust.*, 52:243–256, 1980.

[4] Claude Sauter, Fermin Otálora, José-Antonio Gavira, Olga Vidal, Richard Giegé, and Juan Ma. García-Ruiz. Structure of tetragonal hen egg-white lysozyme at 0.94Å from crystals grown by the counter-diffusion method. *Acta Crystallographica Section D*, 57(8):1119–1126, Aug 2001.

[5] Gerhard Fritz and Alexander Bergmann. Interpretation of small-angle scattering data of inhomogeneous ellipsoids. *J. Appl. Crystallogr.*, 37:815–822, 2004.
